# Printed sensing human-machine interface with individualized adaptive machine learning

**DOI:** 10.1126/sciadv.adw3725

**Published:** 2025-09-10

**Authors:** Guohui Wang, Yao Tang, Xinran Luo, Shengdi Lu, Yiru Zhou, Yi Lu, Guangyang Sun, Pei Liu, Jiayu Ning, Hua Jiang, Ke Hu, Hongzhen Liu, Wenqi Song, You Yu

**Affiliations:** ^1^ School of Biomedical Engineering, ShanghaiTech University, Shanghai, China.; ^2^ State Key Laboratory of Advanced Medical Materials and Devices, ShanghaiTech University, Shanghai, China.; ^3^ Department of Orthopedics, Shanghai Sixth People’s Hospital affiliated to Shanghai Jiao Tong University School of Medicine, Shanghai, China.; ^4^ Tencent Robotics X, Shenzhen, Guangdong, China.

## Abstract

Developing intelligent robots with integrated sensing capabilities is critical for advanced manufacturing, medical robots, and embodied intelligence. Existing robotic sensing technologies are limited to recording of acceleration, driving torque, pressure feedback, and so on. Expanding and integrating with the multimodal sensors to mimic and even surpass the human feeling is substantially underdeveloped. Here, we introduce a printed soft human-machine interface consisting of an e-skin–enabled gesture recognitions with feedback stimulus and a soft robot with multimodal perception of contact pressure, temperature, thermal conductivity, and electrical conductivity. The sensing e-skin with adaptive machine learning was able to decode and classify the hand gestures with re-wearable convenience and individual’s differences. The soft interface provides the bidirectional communications between robotics and human bodies in the close-loop. This work could substantially extend the robotic intelligence and pave the way for more practical applications.

## Teaser

A printed soft human-machine interface enabled gesture recognitions and multimodal perception.

## INTRODUCTION

Flexible electronics are increasingly prevalent in soft human-machine interface applications, which refer to processes through which humans interact and communicate with the outside, especially with computers, robots, and prosthetics (*1*–*5*). The human-machine interface focuses on usability, accessibility, and learnability, ensuring that the interface is intuitive and straightforward (*6*–*10*). Recently, the developments of flexible electronics have enabled sensing devices of soft electronic skins (e-skins) on the body, which can recognize and interpret aspects of vital signs, physical actions, and physiological signal acquisitions like electroencephalography and electromyography for decoding intentions (*11*–*13*). Furthermore, the operation of human-machine interface has developed from single way manipulation to bidirectional communication, adding from multimodal robotic perceptions of tactile sensing, material recognition, and chemical hazard detection (*14*–*17*). Delicate sensing abilities on both sides play vital roles to address the growing demand for the efficient interactive system (*18*–*20*).

Because of the high stretchability and conformability, e-skin as an ideal part of interactive interface has demonstrated an extensive spectrum of applications in embodied intelligence, virtual and augmented reality, surgical robot, service robot, and rehabilitation medicine (*21*–*27*). Despite such promise, several challenges are still remaining for the efficient operations. Although various artificial intelligence methods have already been developed for the classification of physiological signals, only a few have shown ideal performance. Transformer-based approaches tend to be time-consuming, resource intensive, and dependent on complex pretraining procedures (*28*). Recurrent neural networks are good at modeling temporal dependencies but less effective for local patterns (*29*). Fully convolutional networks typically ignore structural features due to their flattened input representation (*30*). While convolutional neural network (CNN) can perform particularly well due to their ability to efficiently capture localized features, operate with low latency, and maintain robustness to signal variability (*31*, *32*). In addition, the lack of low-cost, large-scale manufacture methods, combined with the absence of multimodal sensing for materials recognition, has hindered the widespread adoption of close-loop sensing human-machine interface (*33*–*36*).

**Fig. 1.**
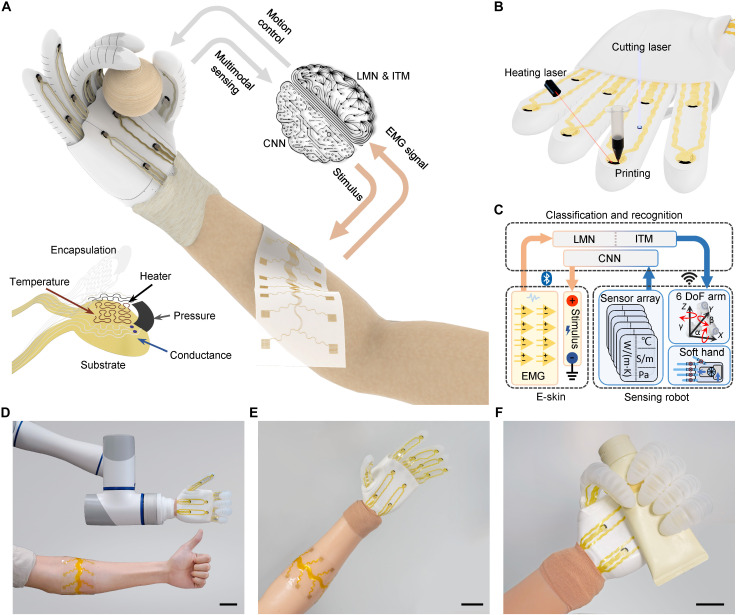
Human-machine interface with individualized adaptive machine learning. (**A**) Illustration of the bidirectional interface with linear mapping net (LMN), inception time modal (ITM), and compact convolution neural network (CNN). Inset, a multimodal sensing array. (**B**) Schematic of fabricated processes for soft sensing robotics. (**C**) Schematic diagram of the human-machine interface with close-loop signal flow between e-skin and sensing robot. (**D**) Photograph of the sensing robot with multimodal sensor arrays. (**E** and **F**) Photographs of e-skin attach on the surface of the arm and soft robot as prosthetic hand (E) to grasp an object (F). Scale bars, 5 cm.

To address these challenges, we propose a printed human-machine interface, consisting of an e-skin for surface electromyography (sEMG) acquisition and stimulus feedback, a sensing soft robot with multimodal tactile perceptions, and machine learning algorithms for gesture classification and material recognition (Fig. 1A). A mass-productive and integrated printing technique was used to fabricated continuous soft bioelectronics of multimaterial and high-density sensor arrays, which consist of direct-ink printing, infrared laser, and cutting laser (Fig. 1B). A linear mapping net (LMN), incorporating inception time model (ITM) was developed to adaptively classify eight channels sEMG signal, which exhibit variability due to individual’s differences and placement inconsistencies (*37*). The soft sensing robot, controlled by the e-skin, functioned as a safe, compliant grasping, and multimodal sensing interface. A CNN processed the sensing data and generated stimulus commands for the e-skin. This allowed for bidirectional communication of gesture classification and robotic perception with the adaptive machine learning enhanced interface (Fig. 1C). This adaptive human-machine interface lays the foundation for a wide range of robotic perception applications and offers a promising direction for the interactive robotic intelligence.

## RESULTS

### Design and print of soft human-machine interface

The sEMG signals were collected from the e-skin on the human body, and the data from hand gestures were decoded and classified into collaborative robotic controlling commands. The sensing robot with multimodal sensor arrays followed the hand gesture through the neuromuscular signals, while the perception of the robot was synchronized through stimulus electrodes on the e-skin to form a closed-loop human-machine interface (Fig. 1D). To enhance the versatility for medical applications, the soft robot integrated with human-machine interface was assembled as the prosthetic hand with feeling for disabled by touching and obtaining the object recognitions (Fig. 1, E and F).

**Fig. 2.**
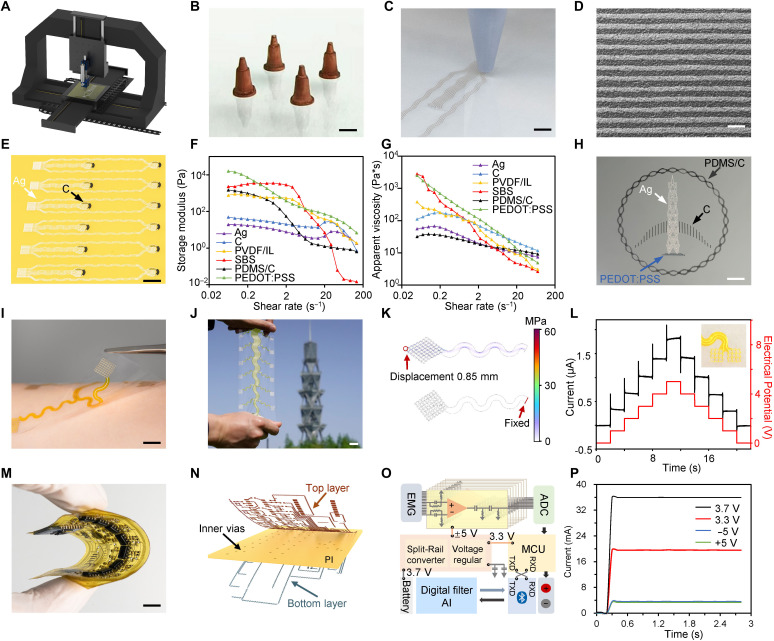
Print and assembly of soft human-machine interface. (**A**) Schematic of the three-axis motion platform integrated with a dispenser. (**B** and **C**) Photographs of laser engraved high resolution nozzle (B) and pattern printing with the dispenser (C). Scale bars, 2 mm. (**D**) SEM image of high resolution printing lines. Scale bar, 100 μm. (**E**) Optical image of printed sensor array. Scale bar, 1 cm. (**F** and **G**) Storage modulus (F) and apparent viscosity (G) characterization of printable inks. C, carbon; PVDF, polyvinylidene fluoride; IL, ionic liquid; SBS, styrenic block copolymers; PDMS, polydimethylsiloxane. (**H**) An emblem pattern printed by four kinds of inks. Scale bar, 5 mm. (**I** and **J**) Photographs of the e-skin attached on a human subject (I) and under mechanical deformation (J). Scale bars, 1 cm. (**K**) Simulation result of von miss stress distribution of sEMG electrode under 0.85 mm displacement. (**L**) Current response of feedback stimulation electrodes on the skin along the applied potential ranged from 0 to 5 V. (**M** and **N**) Photograph (M) and schematic (N) of soft circuit board for sEMG signal acquire and transmission. Scale bar, 5 mm. (**O**) Block diagram of the soft circuit including power supply, stimulus electrodes and Bluetooth module. ADC, analog-digital convert; MCU, microcontroller unit; TXD, serial for transmit data; RXD, serial for receive data. (**P**) The currents of soft circuit board under different voltage supply.

Enabled by inks of multiple materials and programmable path planning, the soft human-machine interface was printed by a precision three-axis moving system integrated with a pneumatic dispenser (Fig. 2A). Tiny nozzles of the dispenser were engraved by a 355-nm ultraviolet laser with multistep processing, the inner diameter could reach 30 μm ultimately (Fig. 2B and fig. S1). The nozzle was assembled with a 3D printing plastic syringe and a plunger (Fig. 2C and fig. S2). The integrated printed system facilitated various advanced capabilities including high resolution, high manufacturing speed, and complex layer alignment. The printed line resolutions could reach 40 μm in width and the patterns showed smooth edges and reproducible conductivities of low variance (Fig. 2D and fig. S3). Different kinds of inks were aligned and printed to fabricate the large-scale multimodal sensor arrays (Fig. 2E). Rheological measurements of diverse inks clearly proved the printability with shear thinning properties (Fig. 2, F and G). Wettability characterization showed that the prepared inks would stay on the substrate with obtuse angle (fig. S4). Especially, polydimethylsiloxane/carbon (PDMS/C) and poly(3,4-ethylenedioxythiophene) doped with polystyrene sulfonate (PEDOT:PSS) ink showed the high aspect ratio on polyimide (PI) substrates, that allowed the capability of overhanging structure. To demonstrate high printing quality with complex materials, an emblem pattern was printed of silver ink, carbon ink, PEDOT:PSS ink, and PDMS/C ink (Fig. 2H and fig. S5).

To realize soft human-machine interaction scenario, an e-skin enabled sensing with stimulus was attached for acquiring sEMG signals and giving feedbacks (Fig. 2I). The soft e-skin was fabricated on ultrathin PI substrate by a series of printing process, laser cutting, and encapsulation coating (figs. S6 and S7). The e-skin exhibited excellent transparency and mechanical flexibility (Fig. 2J). To reduce the strain and achieve uniform strain distribution during mechanical deformation, the e-skin pattern was designed with serpentine structures. The average stress of the sensing electrode was still below 3.3 MPa under 0.85 mm tensile displacement (Fig. 2K). The laser cutting process of removing excess substrates enabled the creation of serpentine patterns, resulting in the flexibility and stretchability (fig. S8). Moreover, a stimulus module was printed at the side of e-skin. While different characteristic voltages were applied, various epidermal stimulation effects could be achieved (Fig. 2L). On the basis of programming applied voltages, the stimulus module could send different codes through the interface (fig. S9).

To cooperate the data collection with the soft e-skin, a bilayer flexible circuit with wireless transmission was fabricated and connected (Fig. 2M and fig. S10). The flexible circuit comprised two layers of printed silver circuits and a middle layer of PI (Fig. 2N and fig. S11). The inner vias serving as pathways for both silver layers were cut through by laser, then fulfilled with silver ink (figs. S12 and S13). With the encapsulation of PDMS, the circuit had high stability during the long-time operation and storage. There were eight series of instrumentation amplifiers with band-pass filters, corresponding to the eight-channel sEMG electrode (Fig. 2O). The voltage output was converted from 3.7 to ±5 V to power the amplification circuit. In addition, a voltage regulator chip was incorporated to ensure a stable 3.3 V output for the microcontroller unit (MCU) and Bluetooth module (Fig. 2P). The analog-to-digital converter within the MCU transformed the analog sEMG signals into digital data, which were then wirelessly transmitted. The circuit was capable of generating 5 V stimulation based on the MCU commands, enabling a closed-loop sensing e-skin system.

### Adaptive machine learning for real-time gestures classification

**Fig. 3.**
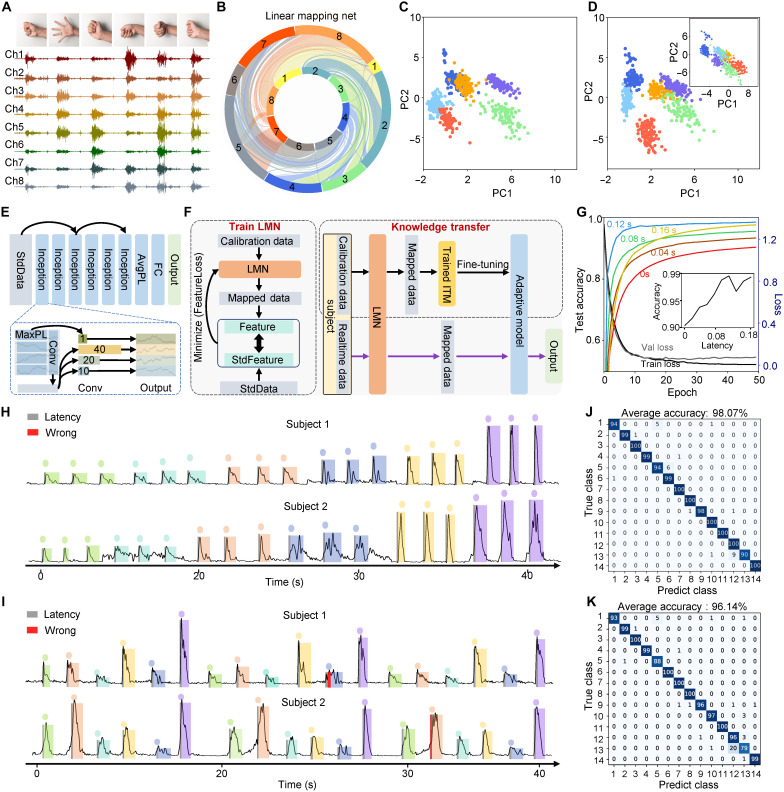
Individual adaptive machine learning model for sEMG data analyze. (**A**) sEMG signals of eight channels from six hand gestures. Inset, gestures from left to right are fist, release, up, down, left, and right. (**B**) Illustration of linear mapping net (LMN) change the eight channels weight. (**C** and **D**) Data distribution by principal components analysis from mapping target subject (C) and a human subject after LMN process (D). Inset, initial data distribution of the subject. (**E**) Inception time model (ITM) for adaptive machine learning. Conv, convolution layer; MaxPL, max pooling; AvgPL, average pooling; FC, fully connect layer. (**F**) Adaptive process with calibration data and StdData by training LMN following knowledge transfer of machine learning model. StdData, standardized dataset. (**G**) Relationship of epoch and test accuracy with different latency in data preparation. Inset, relationship between the latency and accuracy. (**H** and **I**) The sEMG signals and real-time gestures prediction from different subjects. Colored dots indicated prediction time point. (**J** and **K**) Classification results of 14 gestures with the adaptive machine learning model from a pretraining subject (J) and a subject with limited gesture repetitions (K).

The high accuracy of recognition, quick response, and broad individual adaptability were of importance for the human-machine interface (*38*, *39*). A deep neural network system was used to classify various gestures based sEMG signals. Here, the eight-channel sEMG acquiring e-skin was set on the subjects’ forearms with six-gesture test first (Fig. 3A). During the creation of dataset, multiple segments of about 5-min repetitive actions were divided into two parts to avoid cross sampling: the first 4 min and the last 1 min. The first segments are sampled using a sliding window, and the last segments of data were used as test set, and did not participate in training or validation process. Because of sEMG signal variation from individual’s differences and placement inconsistencies, an adaptive machine learning model was proposed that contained LMN and knowledge transfer strategy. The LMN was devised as the initial strategy to adjust the weight of eight-channel signals (Fig. 3B). sEMG signals from another subject were multiplied on the right by a matrix generated from LMN, while signal features were tuned to fit the distribution of target standardized dataset (StdData) (Fig. 3, C and D)

The ITM is a high-performance time series deep learning model, and it was used to perform the sEMG classification task (Fig. 3E) (*37*). Here, a 1-s time window within a designed latency was used for cropping sEMG data (fig. S14). The classification algorithm needed a certain amount of data to make confident decisions. Thus, latency window was proposed as the time offset from the occurrence of a movement signal used for labeling. With different sizes of kernels in conception block of ITM, the model had strong ability to capture time relate information in different timescales. The residual connections between blocks solved the gradient disappearance problem in traditional CNNs (*40*).

Furthermore, a small size of calibration data from single trial was suitable for ITM to learn a general signal representation without overfitting, which reduced the complex pretraining tasks like masked reconstruction or autoregressive modeling of large model. A new subject was only performing repeated gestures with three times, the trained ITM could be tuned into an adaptive model for different subjects with a few recorded actions (Fig. 3F). In details, the calibration data were used to train the LMN by fit their features of peak amplitude and SD with those of the StdData. Moreover, a FeatureLoss function was designed to iteratively update and obtain the finalized LMN with minimized loss (Supplementary Text). For the knowledge transfer strategy, the finalized LMN was used to process the calibration data, generating corresponding mapped data for fine-tuning the pretrained ITM model. Through this fine-tuning process, an adaptive model tailored to the specific individual’s scenario was obtained. During real-time operation, the finalized LMN was also deployed on incoming real-time sEMG signals, which were subsequently fed into the adaptive model to produce classification results.

**Fig. 4.**
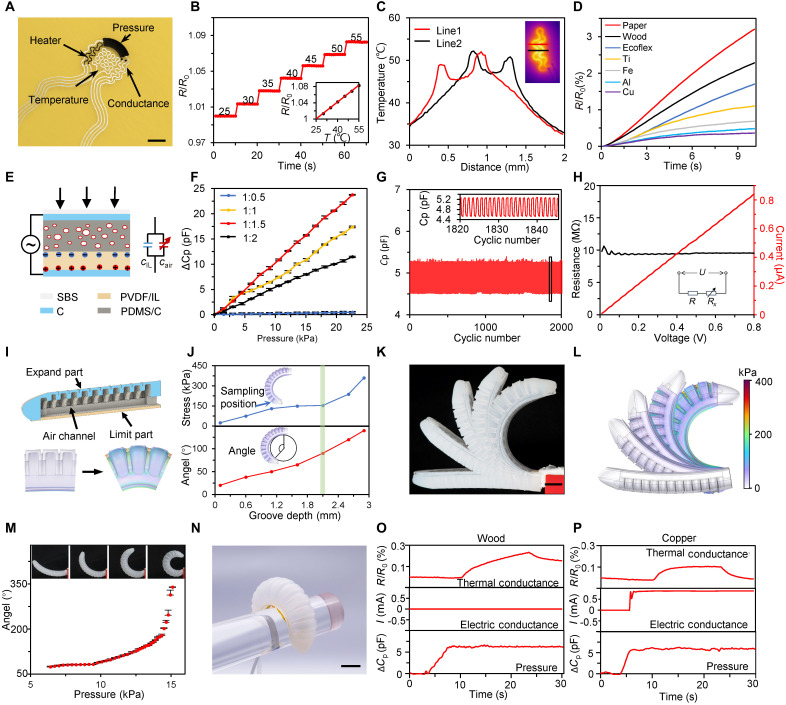
Characterization and evaluation of multimodal sensor array on soft robotic fingers. (**A**) Optical image of a multimodal sensor array. Scale bar, 3 mm. (**B**) Response of temperature sensor. Inset, linear relationship of temperature and resistance change. (**C**) Temperature distribution of a heater along with two lines. Inset, infrared image of the heater. (**D**) Response of thermal conductivity tests for different materials. (**E**) Principle of pressure sensor under alternating current and external press. (**F**) Performance of pressure sensors with different PVDF and IL ratio. (**G**) Long-term stability of pressure sensor. Inset, capacitance signals from 1820 to 1850 cycles. (**H**) Current response of conductance sensor under voltage sweep testing from 0 to 0.8 V. Inset, schematic of conductance test. R, inner resistance; Rx, external object resistance; U, applied voltage. (**I**) Structure design and working mechanism of the soft robot finger. (**J**) Simulated results of different groove depths for balancing the bending angle and stress level. Angle defined by the central angle of a fitting circle from bending shape. (**K** and **L**) Photograph (K) and simulated result (L) of soft finger bending states. Scale bar, 2 cm. (**M**) Relationship between air pressure and bending angle. Inset, Photographs of the soft robot finger with different pressures. (**N**) Optical image of a multimodal sensor array assembled on a soft robot finger. Scale bar, 3 cm. (**O** and **P**) The responses of multimodal sensor array for wood (O) and copper (P).

Low latency was of importance in gesture classification, the balance between the accuracy and the latency should be optimized. In general, a complete sEMG signal piece from single motion encompassed three sections of the initial, the peak, and the end. Therefore, the initial time label was moving discretely from 0 to 0.2 s, the classification accuracy was recorded (Fig. 3G). At 0.1 s latency, the ITM provided 98.33% accurate classifications with the majority of true class probabilities approaching (fig. S15). The real-time classification from gestures could be predicted robustly through the ITM with low latency (Fig. 3, H and I). For the real-time dataset, a fixed 1 s window with 0.1 s latency will still be used to contentiously classify sEMG data and output a gesture result every 0.01 s. It should be classified on the basis of the newest data within the time window of 1 s. Once the beginning of sEMG signal fulfills the latency window, the high accuracy could be achieved. Furthermore, the category of gestures was expanded from 6 to 14 to evaluate the adaptive machine learning model (fig. S16). Unfortunately, the LMN was insufficient to solve the placement inconstancy problem alone. Thus, the fine-tuning process was combined to adjust the model parameters to maintain the high accuracy (fig. S17). The classification resulted in a high overall accuracy of 98.07% on the pretraining dataset (Fig. 3J). To assess the broad individual adaptability, limited gesture repetitions of different participants were used for the transfer learning strategy, the promising outcomes over 93% were achieved (Fig. 4K and fig. S18). The signal-to-noise ratio of the sEMG electrodes maintained for the whole testing processes before and after 20 min of wear during running exercises in distinct environments of different temperature and relative humidity, suggesting that the sEMG system held strong potential for real-world scenario applications (fig. S19).

### Multimodal sensor characterization and application on soft robotics

The multimodal sensor array consisted of a temperature sensor, a pressure sensor, an electrical conductive sensor, and an electrical heater, which was fabricated via serial printing of silver, carbon, polyvinylidene difluoride (PVDF)/ionic liquid (IL), PDMS/C, PEDOT:PSS hydrogel, and SBS inks (Fig. 4A). The sensor array was designed in a wavy structure to provide a certain stretching capability (fig. S20). The temperature sensor was of resistance thermometer in the center of the sensor array. The resistance was changed along with heating and cooling process between 25° and 55°C (Fig. 4B and fig. S21). The temperature sensor exhibited a highly linear response in the range. Then, the temperature sensor was combined with a carbon electrical heater to conduct the thermal conductivity test based on the heat transfer effect of diverse materials. In brief, the heat generated by the electrical heater diffused through the adjacent object, the temperature sensor nearby could detect the changes, and then provide a means of identification by its own property of thermal conductivity (fig. S22). The resistance of the carbon heater was relatively much higher compared to silver interconnects, while it generated a notable heating effect with 10 V applied only on the specified area (Fig. 4C). Several materials were tested with the thermal conductive sensor combination, and the higher the thermal conductivity, the lower the temperature increase (fig. S23). Under the certain setup of the sensor combination, the response of temperature sensor showed differences among various materials (Fig. 4D). The pressure sensor was a capacitive type sensor with printed multiple layers, wherein the functional layers were PDMS/C with embedded bubbles and PVDF incorporating with IL (Fig. 4E). The presence of microbubbles enhanced the resilience and capacitance change (fig. S24) (*41*). Simultaneously, the free charges within the PVDF/IL membrane created numerous microcapacitors under the influence of applied electric fields (*42*). As the ratio of PVDF to IL varied from 1:0.5 to 1:2, the sensitivity of the pressure sensor reached the highest of 10.5 pF kPa^−1^ at ratio of 1:1.5 (Fig. 4F). The concentrations of IL should balance the insufficiency of resilience and numbers in microcapacitors as the middle layer, and the pressure sensor demonstrated excellent stability over 2000 consecutive experimental cycles (Fig. 4G). The operating principle of the conductance sensor involved applying a certain voltage between two electrodes, then measuring the current across the object. Because of the great electrical conductivity and high aspect ratio of PEDOT:PSS hydrogel, it was capable to facilitate the sufficient contact with the object surfaces (*43*). For instance, 20 μl of IL (BMIMBF_4_) covering both electrodes was tested with 0.1 V applied, the resulting current is expected to be in the microampere range (Fig. 4H). Detected by the infrared camera, the nearest edge of pressure sensor close to heater and the middle of conductance sensor were heated up by 10° and 4°C amplitude (fig. S25). A pressure sensor was heated in the range of 10°C cyclic 500 times, and capacitance maintained a stable state during test (fig. S26). The sensors exhibited resistance variations below ±1.6% with bending deformations, 3.0% with abrasion, and less than 5% after tensile strains of 1000 cycles for each (fig. S27).

The multimodal sensor arrays were attached on soft robotics, which were designed with elastomeric airbag structures (*44*–*46*). The expand part at the top was composed of Ecoflex elastomer, while the bottom limit layer was made of PDMS (Fig. 4I). Considering the significant difference in the Young’s modulus of the two silicone materials (fig. S28), the soft robotic finger exhibited distinct bending behaviors upon inflation. The soft robotics enabled the sensors to conform tightly to varied and irregular object surfaces to adapt firmly to diverse and irregular object surfaces. For the airbag structures, the simulated analysis indicated that the soft finger achieved a larger bending angle with the groove depth of 2.1 mm under the same pressure. At the same time, the maximum stress in the thinned part remained at a relatively low level of 153 kPa (Fig. 4J). The actual finger bending had matched the simulated analysis well under different pressure inside (Fig. 4, K and L, and figs. S29 and S30). At low pressure level, the bending angle below 180° changed linearly with the pressure inside, then rapidly rise near 360° within 3 kPa pressure increase (Fig. 4M). The soft robotic fingers were operated for over 5000 times by maintaining the stabilities (fig. S31). Besides, a soft finger with two sections of chamber at oblique angles was designed as the twist thumb for the sensing robotic hand (fig. S32) (*47*). Integrated with multimodal sensor array, the sensing robotic finger was inflated to hold and sense cylinder shape objects (Fig. 4N). For the object recognition experiment, the multimodal sensors were sequentially activated. The pressure sensor and the conductance sensor were detecting all the time. Upon the pressure sensor detecting contact, the heater was triggered to facilitate thermal conductivity measurements in conjunction with the temperature sensor. Throughout the process, dynamic testing data from multimodal sensor array were recorded and compiled into a dataset for subsequent analysis. With wood and copper objects, the sensing robotic finger exhibited distinct responses from pressure sensors, electrical conductivity sensors, and temperature sensors, separately (Fig. 4, O and P).

### Evaluation of interactive soft robotic for object recognition

**Fig. 5.**
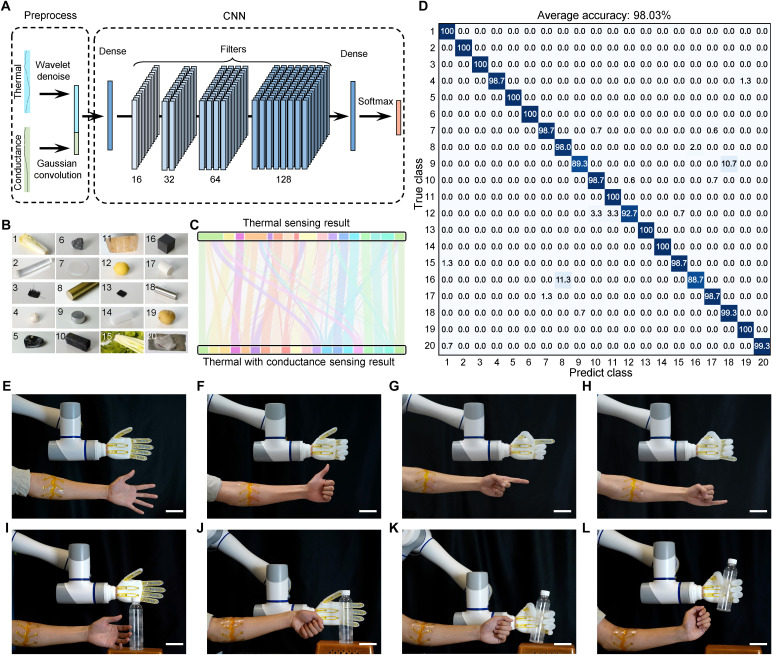
Evaluation of interactive soft robotic hand for object recognition. (**A**) The preprocess and architecture of material recognition algorism with a compact convolution neural network (CNN). (**B**) Images of 20 kinds of objects. The number 1 to 20 represented materials as follow: cabbage, glass, fabric, garlic, single-crystal silicon, polycrystalline silicon, silicone rubber, brass, aluminum, charcoal, pumpkin, lemon, conductive gel, polyethylene foam, celery, graphite, acrylonitrile butadiene styrene (ABS) plastic, stainless steel, potato, and lamb, respectively. (**C**) Sankey diagram of recognition results change from just using thermal conductivity sensing to thermal with conductance sensing. (**D**) Recognition result involved 20 materials by recognition algorism. (**E** to **H**) Photographs of the soft sensing robot controlled by the human-machine interface with adaptive machine learning. Scale bars, 5 cm. (**I** to **L**) Time-lapse photographs of the soft sensing robot grasping a bottle object. Scale bars, 5 cm.

By integrating the printed sensors onto a soft robotic hand, the electrical conductivity was first introduced into the object recognition to developed the system feeling capability combining with simultaneous measurement of thermal conductivity. By combining the capabilities of electrical and thermal conductivity sensor arrays, a neural network was used for object recognition (Fig. 5A). The thermal and electrical conductivity data were processed to ensure optimal input for the neural network, with tailored denoising and smoothing techniques. In contrast to sEMG signals containing rich high-frequency components and complex temporal fluctuations, multimodal sensor data exhibit more gradual and monotonic changes, like clearly rising and platform. The preprocessed data were concatenated and then input into a compact CNN. In details, thermal signals were preprocessed using wavelet transforms to suppress noise, while conductivity signals were smoothed by gaussian convolution to reduce mechanical artifacts. Following normalization by SD, a fourth-root transformation was applied to compress conductivity variations, before resampling and concatenation for network input. Ablation studies confirmed the effectiveness of each step, with the removal of wavelet denoising, Gaussian convolution, and compression causing accuracy declines of 1.83, 0.96, and 6.13%, respectively. The preprocessed data were then resampled, concatenated, and input into the recognition network. The network started with a fully connected input layer of width 128, and followed by a sequence of 3 × 3 convolutional layers featuring 16, 32, 64, and 128 kernels accompanied by batch normalization and ReLU activation. A fully connected layer with 256 units and ReLU activation was used for dimensionality reduction. Last, the output stage comprised a fully connected layer, a SoftMax layer for class probability computation, and a final classification layer. There were 20 kinds of objects involved to evaluate the interactive robotic recognition, include materials of metal, semiconductor, plastic, food, and so on (Fig. 5B). Thermal conductivity data alone achieved an average material identification accuracy of only 63.99% (fig. S33). While additional dimensional conductance data were incorporated, the overall accuracy increased to 98.03% by correctly reassigning diverse objects (Fig. 5, C and D). The compact recognition algorithm could be further evaluated with 2.089 ms in an embedded system with 8-GB memory (fig. S34). This design was not only a combination of sensing methods but also an engineered multimodal mechanism that leveraged the fundamental physical properties of the materials, representing an improvement of accuracy and recognition numbers (table S1). For the object recognition task, it was strictly derived from held-out test folds using a nested fivefold cross-validation strategy. This approach separated the hyperparameter tuning from the final performance evaluation, ensuring that the model was not evaluated on data it has seen during training (*48*, *49*). Such a setup provided a robust safeguard against overfitting and yielded a more reliable estimate of generalization performance. In compare, several machine learning models commonly used in classification tasks of ITM, support vector machine, gaussian mixture model, random forest, and linear discriminant analysis had been tested with accuracies of 98.63, 87.89, 83.67, 90.17, and 87.60%, respectively (fig. S35). Although ITM showed relatively higher accuracy with 0.6% increase, the training time increased by nearly four times, making the trade-off less favorable in practical scenarios.

At last, multimodal sensor arrays were fitted on the interactive sensing robotic hand of five different soft fingers and a hard palm integrated with solenoid valves inside. The robotic hand had a maximal 14.04-W energy consumption corresponded that all six valves and an air pump turned on (fig. S36). The robotic hand was assembled on a collaborative robotic arm to form a complete interactive sensing robot, the robot could follow and perform movements and finger gestures wirelessly, which were collected and decoded from the human subjects wearing the soft e-skin (Fig. 5, E to G). Moreover, the sensing robot could be controlled to perform simple grasping tasks and object recognition (Fig. 5, H to K).

### Evaluation of the human-machine interface on upper limb disabled

**Fig. 6.**
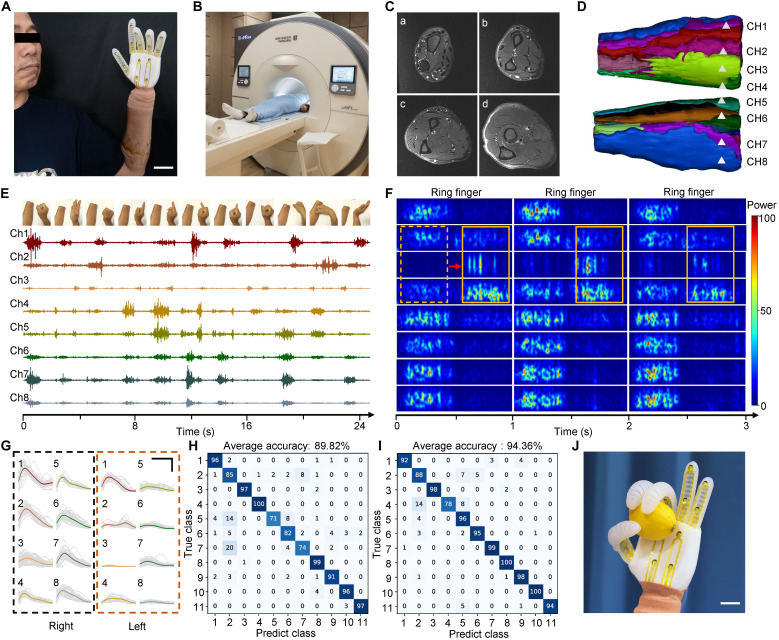
Evaluation of the human-machine interface for prosthetic application. (**A**) Photograph of the human-machine interface as prosthetics wearing on a subject after amputation surgery. Scale bar, 4 cm. (**B**) Optical image of a MRI scanner for soft tissue identify. (**C**) MRI image of the subject’s left forearm. a to d, the distances are 5.7, 12, 16.5, and 20.7 cm from the elbow. (**D**) Reconstruct model with the eight-channel sEMG electrode base on the MRI images. (**E**) sEMG signals of eight-channel from 11 hand and finger gestures. Inset, (left to right) fist, release, up, down, left, right, thumb finger, index finger, middle finger, ring finger, and little finger. (**F**) Time-frequency plot of ring finger gestures for three times. The frequency of each dependent signal was from 0 to 300 Hz. Inner rectangle and arrow indicate the time delay phenomenon. (**G**) The average intensities of sEMG signals in eight channels from both arm of the amputee. Inset scale bar, horizontal axis represents 1 s, vertical axis represents 0.8 mV. (**H** and **I**) Classification matrix of 11 gestures with the adaptive machine learning algorithm from the subject with 20 times (H) and limited 5 times gesture repetitions (I). (**J**) Photographs of the soft sensing robot as prosthetic hand to grasp a lemon. Scale bar, 5 cm.

The sensing human-machine interface with soft robot was used as a smart prosthetics with controlling and feeling for disabled, for further versatility on medical applications. A subject after the amputation surgery of the left arm has worn the e-skin and the sensing robot as the sensing prosthetics (Fig. 6A). To identify the degeneration and absence of tendons and muscles after the surgery, both arms of the amputee were scanned with the magnetic resonance imaging (MRI) (Fig. 6B). It showed that the muscle structures were integrity, and extensive edema existed between muscles and intermuscular septa compare to the right hand (Fig. 6C and fig. S37). The left arm was then reconstructed based on the MRI data, to identify the positions of the eight-channel sEMG electrodes (Fig. 6D).

The subject performed 11 actions of single-finger gesture and whole-hand movements with both right and left arm at the same time, which was more convenient and precise to activate the related muscles for controlling the nonexistent hand (Fig. 6E). In contrast to sEMG time frequency plots of the right arm, those on the left exhibited misalignment phenomenon and time latency at certain channels (Fig. 6F). For instance, the third channel on the left arm around brachioradialis showed about 0.42-s asynchrony of the ring finger gesture, and the sixth channel extensor carpi ulnaris also had about 0.33-s misalignment at the index finger gesture (fig. S38). Instead, the signal latency of gestures, with most muscles recruitments at the same time like grasp and release, could rarely been obtained. In contrast, residual muscles compensated to perform movements without the visual and tactile feedback, as the damage of the muscular and neural architecture. This reorganization yields muscle activation patterns that typically lack the stability observed in intact limbs, resulting in temporal latency variations and inconsistency (*50*, *51*). Besides the time latency, the signal intensities at the left arm were significantly lower over 40% on average than those on the right as well (Fig. 6G). The weak signals at certain channels might be attributed to abnormal conditions of nerves and muscles degeneration after the surgery (*52*–*54*). Despite the latency and decrease of the sEMG signals, the gestures from the subject could still be recognized by the adaptive machine learning and all the data process about adaptive machine learning are totally same as normal condition. For interactive robotic controlling, 50 times of each gesture were recorded and an overall mean accuracy of 94.36% was achieved (Fig. 6H). Considering the rapidity and convenience, the adaptive machine learning approach proved a reliable solution to the rewearability challenge of e-skin. An overall mean accuracy of 89.82% was obtained with another trail of five repetitions (Fig. 6I). The e-skin for amputees could be operated with fewer time of positioning and wearing, while it proved the versatility with transfer learning strategy. The system successfully captured and interpreted sEMG signals, enabling them to complete a series of control tasks and stimulus feedback. Last, it demonstrated that the assisted grabbing and feeling from prosthetics could be accomplished with the sensing robot in real-world circumstances (Fig. 6J). By incorporating the electromyography recording along with the object recognition multimodal sensors, the soft human-machine interface could facilitate the manufacture and operation of interesting prostheses that bring into the close-loop of feeling and controlling.

## DISCUSSION

Here, we have described a mass-producible soft human-machine interface with individual adaptive machine learning. The human-machine interface is formed by an e-skin–enabled sEMG sensing system with feedback stimulus and a soft robotic hand functioned with multimodal sensor arrays. In this work, we integrated a printed system with direct ink writing and laser cutting to fabricate soft e-skin and flexible multimodal sensor arrays. The printed multimodal sensor arrays were attached on soft robots and performed the measurements of contact pressure, temperature, thermal conductivity, and electrical conductivity. The safety and fit of soft robot is critical for human-machine interaction and prosthetic applications, aiding the restoration of perception functions. Enabled by consistent mass production, its modular and scalable design allows to serve as a foundational building block for complex systems, and the sEMG sensing e-skin with adaptive machine learning was able to decode and classify the hand gestures with rewearable convenience and limited repetition as well. The integration of the soft interface provides the bidirectional information communications between robots and human bodies in close loop. The sEMG sensing e-skin ensures stable contact with the soft human skin to record neuromuscular signals for the complex gesture recognitions. We introduce a compact neural network as the transfer learning strategy to achieve the high accuracy, low latency, and individual adaptability, instead of multiplexed pretraining tasks and large computility demand in the large model. On the other hand, the sensory modality combination of thermal and electrical conductivity on soft robots allows for accurate recognition of diverse objects.

This interactive loop of robotic feeling and controlling represents an attractive approach for the embodied intelligence, which not only endows robots with powerful perceptual capabilities but also facilitates the interpretation based on multimodal sensing modalities. It underscored the fundamental role of perception in the robotic intelligence, the robots should accurately and reliably perceive the surroundings to make optimal decisions within an unknown scenario. Moreover, by integrating the new kinds of sensors and cooperating with computer vision, this technology could substantially extend future robotic intelligence and pave the way for more practical robotic applications.

## MATERIALS AND METHODS

### Materials

All chemicals were used as received without further purification. PVDF–hexafluoropropylene (HFP; molecular weight, ~400,000) was purchased from Sigma-Aldrich. Silver ink and carbon ink were purchased from Shenzhen Saiya Electronic Paste Co. Ltd. PDMS (Sylgard 184) was purchased from Dow Inc. 1-Ethyl-3-methylimidazolium tetrafluoroborate (BMIMBF_4_; 14 mS cm^−1^), sodium bicarbonate was purchased from Aladdin. Carbon black (30 nm) was purchased from Cool Chemical Science and Technology (Beijing) Co. Ltd. Potassium chloride, toluene, acetone, DMSO, silicon dioxide (7 to 40 nm) were purchased from Sinopharm Chemical Reagent Co. Ltd. SBS (D1118E) was purchased from Karton. PEDOT:PSS (PH-1000) was purchased from Heraeus Electronic Materials.

### Preparation and characterization of print inks

To prepare the PDMS/carbon ink, 0.005 g of carbon black, 0.025 g of NaHCO_3_, and 0.4 g of IL were mixed with 9 g of PDMS, and then magnetically stirred for 2 hours at room temperature to ensure the particles were homogeneously dispersed in PDMS. Then, hydrophilic 0.3 g of silicon dioxide was added inside and stirred for 1 hour. At last, 1 g of cure regent was added into the mixture and degassed for 10 more minutes. Two grams of PVDF was first dissolved in 10 g of DMSO while being stirred vigorously at 65°C for 3 hours, and then 2.4 g of IL was added and stirred for 1 hour to form the PVDF/IL ink.

Encapsulation SBS ink was prepared by 2 g SBS dissolved in 9.2 ml of toluene to form 20 wt % SBS ink. For PEDOT:PSS ink, the aqueous solution was stirred vigorously over night at room temperature. Then, the solution was frozen by liquid nitrogen and processed in a freeze dryer for 48 hours. The obtained PEDOT:PSS fibers were redispersed into deionized water to form a 7 wt % solution. The prepared PEDOT:PSS ink was kept at 4°C before use. Silver and carbon ink were used as received.

Rheological characterizations of all the ink were conducted using a rotational rheometer (Haake Mars iQ Air, Thermo Fisher Scientific) with 35-mm-diameter steel parallel-plate geometry. Apparent viscosity was measured by steady-state flow tests with a logarithmic sweep of strain (0.05 to 200 s^−1^). Shear storage modulus (*G*′) and loss modulus (*G*″) were measured as a function of strain via oscillation tests with a logarithmic sweep of strain (0.1 to 100) at 1 Hz shear frequency. All rheological characterizations were conducted at 20°C. Characterization of wettability for all inks on PI surface were taken with a contact angle measurement (OCA15EC GmbH).

### Assembly of 3D ink printing system

A cooper plate of 2 mm thickness was laser engraved for fabricating the high-resolution needle (Protolaser U4, LPKF). The cooper plate was first engraved an inner hole with a power setting of 5 W, a frequency of 40 kHz and a mark speed at 100 mm s^−1^, the first layer of the inner hole was set to a diameter of 0.6 mm with a focus offset of 0 mm and 15 repetitions. The second layer featured a diameter of 0.4 mm with focus offsets from 0.2, 0.5, to 1.5 mm with repetitions of 15, 20, and 40, separately. Focus offsets and the repetitions of each process of layer were adjusted accordingly. Then, it was flipped and engraved as cone shape by three steps from different diameters of 1.5, 1, and 0.6 mm with 20 repetitions. The cap of the needle was designed with a diameter of 2 mm, leading to the establishment of the third layer with 1.5 mm focus offsets and 40 repetitions (fig. S1). The shell of syringe was produced of photosensitive resin by a 3D printer (Saturn 4 Ultra, Elegoo). The copper needle was sticked into the syringe with glue (fig. S2).

A three-axis motion platform (AUS-Precision) was used to hold the syringe and camera system as the 3D position system. The camera system was designed aiming at the alignment of multiple printing layers. The printing syringe was assembled with an air tube that connects to a normally closed solenoid valve and a normally open solenoid valve to control the pressure on and off. The designed patterns were converted into the controlling codes through SPiiPlusSPC software (ACS Motion Control). The moving velocity of the printing system ranged from 1 to 30 mm s^−1^ during the tasks, and the air pressure of the syringes ranged from 0.1 to 0.6 MPa.

### Fabrication and characterization of the soft human-machine interface

The fabrication process of e-skin with eight-channel sEMG and stimulating electrode was illustrated in fig. S7. In brief, silver interconnects were printed through the 3D ink printing system on PI substrates, and the substrate was removed along with the designed pattern by laser cutting. For encapsulation, PDMS with a ratio of 10:1 was spin coated with 500 rpm for 30 s on a silicon wafer at first (WS650, Laurell). One-millimeter-thick polyethylene terephthalate pieces were used as masks on electrodes and pins, which should be exposed. Then, the printed sEMG electrodes were spin coated with 500 rpm for 30 s to isolated the interconnects. The circuit patterns were printed onto both sides of a PI substrate using conductive ink. Then, designed inner vias serve as pathways between the both sides of circuit layers are cut through by laser (Protolaser U4, LPKF). Silver ink was printed around the inner vias on both sides to establish electrical connectivity through the inner vias. After the required electronic components were soldered, two layers of PDMS (10:1 wt %) were coated on both sides to isolate the flexible circuit board. Conductive paste was used to connected to the acquisition electrodes and the circuit to form the soft human-machine interface. For the simulation of sEMG electrode, the fundamental setup involved using the membrane module, setting material as PI (0.1 mm; Young’s modulus, 200 MPa), add spring foundation to the model, and use triangular mesh. Specific displacements were applied on the model boundaries to induce deformation and subsequently, the resulting stress distribution was obtained.

The wireless bilayer flexible circuit facilitated the real-time transmission of sEMG signals and the generation of electrical stimulation on the skin. To verify sEMG feedback function, an electrochemical workstation (842d, Shanghai Chenhua) applied programmed voltage (0 to 5 V) on stimulation electrodes and measure current on the skin. A voltage regulator (TPS73233) and a split-rail converter (TPS65133) provided the necessary power supplies, delivering 3.3 and ±5 V to the analog and digital circuits, respectively. Eight instrumentation amplifiers (INA118) were used to amplify the sEMG signals by a factor of 2000, with passive filters applied to attenuate noise below 10 Hz and above 500 Hz for each channel. The system sampled at 1 kHz, which was four times higher than the primary frequency of the sEMG signal. Analog-to-digital converters integrated in the STM32F103C8T6 microcontroller (MCU) were used to capture the eight-channel analog signals in DMA mode. These data were transmitted via the serial peripheral interface to a Bluetooth low energy (BLE MX-22) module, which sent the data to a PC and received stimulation feedback command. sEMG signal acquisitions with dynamic movements were recorded before and after 20-min exercises under two distinct environmental conditions of 16°C, 62% relative humidity and 25°C, 75% relative humidity. To realize electrical stimulation, two General Purpose Input Output (GPIO) pins of MCU connected functional and reference electrodes were configured in open-drain mode to deliver 5 V output. Upon receiving a signal indicate, the MCU triggered electrical stimulation with defined duration.

### Adaptive machine learning for real-time gestures classification

For the initial sEMG calibration signal acquisitions, a standard setting process was taken. First, a subject positioned the right forearm straightly in the front while the right palm is vertically facing left. The subject’s skin was cleaned by alcohol swabs. Then, the channel electrodes close to the connect pins as called Ch1 was aligned with the top side of the forearm, corresponding to the extensor carpi radialis. Last, the sEMG electrode array was naturally wrapped and fitted on the skin, wherein the Ch8 corresponded the brachioradialis. Then, the subject was performing 14 hand gestures sequentially with each discrete movement repeated 100 to 200 times with participants instructed to hold each gesture for about 1 s. The 14 gestures were fist, unfold, up, down, left, right, thumb finger, index finger, middle finger, ring finger, little finger, and finger gestures of two, three, and four, respectively. The circuit sampled at 1 kHz, and we used a sliding window to generate training and validation data points every 0.01 s. Data were transferred via Bluetooth and virtual serial port with 1000 Hz sampling frequency. To enhance data quality, several digital filters were applied, including a band-pass filter (10 to 245 Hz) and a band-stop filter centered around 50 Hz to remove noise. A 1 s time window was used to create a StdData, maximizing the information content while minimizing potential interference. Sliding SD algorithm was used for labeling, distinguishing rest from active states, ensuring a balanced dataset to prevent overfitting.

The ITM consisted of six inception modules, each with four parallel paths. Three paths used convolutional layers with kernel sizes of 10, 20, and 40 to capture temporal features at different scales. The fourth path performed identity mapping to retain raw input features. The outputs of these paths were concatenated and passed through a 1 × 1 convolutional layer to reduce dimensionality and integrate multiscale features. Residual connections were used between modules to address the vanishing gradient problem, ensuring stable training and enabling the model to scale effectively. A bottleneck layer compressed the input before convolution operations, optimizing computational efficiency and reducing model complexity. For the small-dataset transfer learning process, a new subject performed 14 gestures, each repeated three times with a 2-s interval, resulting in a total of 42 actions. A 0.01 s sliding window was applied to sample these recordings into a small dataset. Then, both calibrated features and StdData features were calculated and used to train the LMN. The data were automatically segmented and labeled for training the LMN. After completing the LMN process, a two-step fine-tuning strategy was applied. In the first step, the calibration data were mixed with the StdData at a ratio of 1:5 and trained for 50 epochs with a learning rate of 0.001. In the second step, the mixing ratio was adjusted to 1:1, and training continued for another 50 epochs with a reduced learning rate of 0.0005. In the real-time classification system, the recording circuit continuously transmitted data. Every 0.02 s, a new 2-s data segment was input into the trained model, with output generated in less than 0.02 s.

### Fabrication and characterization of robotic hand

The multimodal sensor arrays on soft robotics were fabricated via serial printing of silver ink, carbon ink, PEDOT:PSS ink, PVDF/IL ink, and PDMS/carbon ink according to the designed pattern. Then, the PDMS/carbon layer was heated to generate uniform bubbles with 808-mm infrared laser (MDL-H, CNI). Another layer of carbon ink on the pressure sensor followed by the encapsulation SBS layer on the whole sensor arrays were printed with the 3D ink printing system. Last, the blank parts of substrates were removed by laser cutting with a frequency of 45 kHz, a power output of 5.58 W, and processing speed of 500 mm s^−1^.

The robotic fingers were made by scalable mold method (fig. S32). There were two kinds of molds, the ones of polylactic acid (PLA) were manufactured with 0.2-mm nozzle of the 3D printer (X1C, Bambu), the other aluminum plates were processed by a five-axis computer numerical control machine (DMU50, DMG Mori). Ecoflex 00-50 was slowly poured and into the PLA base mold with 15-min degassing, the other part of the mold was then fit together with the former mold. The mold with rubber inside was solidified in 40°C for 1 hour. The PLA molds were then placed into the aluminum plates together with PDMS (10:1 wt %), where the fingers were treated at 60°C for 2 hours. Subsequently, some silicone adhesives were used to affix the robotic fingers on the palm, which was designed and fabricated of photosensitive resin by the 3D printer (Saturn 4 Ultra, Elegoo). Inside the palm, the silicone rubber fingers were connected with six solenoid valves, which consist of an open valves and five closed ones. The solenoid valves were controlled by a designed board of a MCU (Arduino Nano ESP32) and pressure sensors (ZGZP6847A, Diymore) with capability of autonomous pneumatic regulation. A compact vacuum pump (KVP04, Kamoer) was assembled to generate the air pressure. For bending test, the intake valve was closed and it maintain the state for 4 s before opening the exhaust valve, while the air pressure reaches the preset value. For the simulation of robotic fingers, the fundamental setup involves using the solid mechanics module, defining boundary condition and material (Ecoflex and PDMS) model as hyperelastic, and using free tetrahedral meshing (fig. S29). The three-order Ogden model was selected for fitting the material machinal properties (fig. S28). The parameters used in the simulation are as follows. For Ecoflex, the shear moduli (μ) and exponents (α) for the three terms were (μ_1_ = 47.20 MPa and α_1_ = 2.086), (μ_2_ = −78.37 MPa and α_2_ = 2.57958), and (μ_3_ = 35.21 MPa and α_3_ = 2.9897). For PDMS, the corresponding parameters were (μ_1_ = 1.97 MPa and α_1_ = 2.911), (μ_2_ = −3.671 MPa and α_2_ = 3.008), and (μ_3_ = 1.74 MPa and α_3_ = 3.096). The inner surface was applied 6 kPa over 10 incremental substeps to observe the deformation outcomes of various depth of groove. The multimodal sensor arrays were fitted on all the fingers and the palm, separately. Concerning to heater simulation, the experiment simulates the time-dependent thermal response of a heater and its PI substrate, modeled as a thermally thin assembly with a 0.15 W volumetric heat source, interacting with a cubic object of 2 cm × 2 cm × 2 cm via a thermal contact interface (considering constriction resistance, conduction, and radiation) and exchanging heat with the ambient environment through convection, all while starting at an initial temperature of 293.15 K and analyzed over 12 s.

The temperature sensor characterization was performed on a hot plate ranging between 25° and 55°C with a programmable power source (DP832, Riglo), and the resistances of temperature sensors were recorded by an impedance analyzer (TH2836, Tonghui). The thermal images were obtained via an infrared camera (248M, Fotric) at the same time. For thermal conductance sensor characterization, a sensor was attached on different materials and was applied voltage 10 s. In this process, the temperature increasing of the object was recorded by the central temperature sensor, where it was changed by the heater electrode nearby. During the test, the voltage of heater electrode was set to generated 0.15 W power for 10 s. The detection for capacitance of pressure sensor (*C*_p_-*G*) and current response of conductance sensor were measured by a parameter analyzer (4200A-SCS, Keithley). For cyclic temperature dynamic testing, the pressure sensor was subjected to a constant load and placed on a hot plat ranging 25° to 35°C using a programmable DC power supply (DP832, Riglo). Surface temperature was monitored by an infrared camera (248M, Fotric). Conductance sensor characterization was performed with 20 μl of IL (BMIMBF_4_) under a voltage linear sweep from 0 to 0.8 V to verification. For object recognition, the conductance sensor was tested with 0.1 V applied. For bending fatigue assessment, the sensors were integrated onto finger to repeated bending deformations. Abrasion resistance was evaluated by applying a rough plane with 0.1 mm protrusions under 3.9 kPa, across a 2 cm stroke. The stretchability of the sensors was tested by applying a tensile strain of 6%, exceeding typical forearm skin deformation during muscle contraction and relaxation. To investigate environmental stability, an accelerated aging test was performed by exposing the printed temperature sensors to conditions of 85°C and 85% relative humidity for 168 hours. The CNN model was deployed on an 8GB Developer Kit (Jetson Orin Nano, NVIDIA), which was equipped with a 256GB NVMe SSD. The system run on the NVIDIA JetPack 5.1 environment, using CUDA 11.4, cuDNN 8.6.0, TensorRT 8.5.2, and PyTorch version 2.0.0.

### Object recognition algorithm by multimodal sensors on finger

For object recognition with multimodal sensors, 20 different kinds of objects were involved. The multimodal sensor array was affixed on a soft robotic finger to be contact with the object surfaces, followed by repeatedly bending motions. For object recognition, the dataset consists of 3000 samples (20 classes × 150 samples per class). The collected data were then analyzed using machine learning methods. The data processing pipeline involved several steps to enhance the quality and interpretability of the sensory information for material recognition. To address domain-specific challenges in the sensor signals, we applied three preprocessing steps before model input. Wavelet denoising was used to suppress high-frequency thermal noise observed in resistance measurements; gaussian convolution smoothed transient conductance spikes arising from contact mechanics rather than intrinsic material properties; and a fourth-root compression mapping mitigated the large dynamic range between highly and weakly conductive objects, preserving intraclass discriminability. All datasets were subsequently normalized by their SDs. In addition, a compressive mapping, using the fourth root, was applied to the conductivity data to better retain discriminative information despite large variations in magnitude. The processed data were then resampled, concatenated, and fed into a recognition network for classification.

The recognition network began with a fully connected input layer of 128 units, followed by sequential 3 × 3 convolutional layers with 16, 32, 64, and 128 kernels. Each convolutional layer was paired with batch normalization and ReLU activation to enhance learning stability and introduce nonlinearity. A fully connected layer with 256 units and ReLU activation further reduced the dimensionality. Last, the output layer consisted of a fully connected layer producing logits, followed by a SoftMax layer to compute class probabilities, and a classification layer to determine the predicted class. Object recognition results are based on offline training.

### Evaluation of human-machine interface

The sensing robotic hand was assembled on a collaborative robot (CR5, Dobot) through a 3D printed connector of photosensitive resin. The control code was written with Python. The sEMG sensing e-skin was synchronously set around a human participant’s forearm after cleaning the skin with alcohol swabs. Every participant was performing symbolic gestures for three times, aiming at learning feature of the algorithm. Results were sent to collaborative robot and sensing robot to conduct arm motion and robotic hand gesture, separately. When the responses were achieved from the multimodal sensors with object recognition, the e-skin could send featured electric stimulations back to human skin.

The validation and evaluation of the sensing human-machine interface were performed of human participants in compliance with all the ethical regulations under protocols (ID Q2022-053) that were approved by the ethics committee at the ShanghaiTech University. Nine participates were recruited from the ShanghaiTech University and the neighboring communities through advertisement. All of participants accepted and signed informed consent before study participation.

### Evaluation of human-machine interface on participant after amputation surgery

An MRI scanner of 3 T (UMR 890, United Imaging) was used to get the soft tissue information from both arms. A T2-weighted imaging method with fast spin echo and fat saturation was used (field of view of 140 mm × 140 mm, 230 × 288 matrix size, 3-mm slice thickness without any gap, reception time = 6100 ms, and echo time = 82.98 ms). Then, T2 image was imported to Mimics software to construct a 3D model. The experiment was conducted 8 weeks after amputation surgery.

Subject’s skin was cleaned by alcohol first before acquire sEMG data. Conductive paste was applied on the sEMG electrode and connected to the acquisition device and circuits. The subject performed gestures simultaneously with both his residual limb and his healthy contralateral hand, while imaginarily for the residual limb. The interval between each action was about 2 s.

The validation and evaluation of the sensing human-machine interface were performed of the human participant in compliance with all the ethical regulations under protocols (ID 2022-053) that were approved by the ethics committee at the ShanghaiTech University. The participate was recruited from the Shanghai Sixth People’s Hospital. Participants accepted and signed informed consent before study participation.

## Supplementary Materials

### This PDF file includes:

Supplementary Text Figs. S1 to S38 Table S1 References
