# Supplementary material for: Printed sensing human-machine interface with individualized adaptive machine learning
Source: Sci Adv. 2025 Sep 10;11(37):eadw3725. doi: 10.1126/sciadv.adw3725 (PMC12422187; doi:10.1126/sciadv.adw3725)
Supplement: Supplementary file 1 — Supplementary Text Figs. S1 to S38 Table S1 References [file sciadv.adw3725_sm.pdf]

Science Advances
AAAS

Supplementary Materials for

Printed sensing human-machine interface with individualized adaptive machine learning

Guohui Wang *et al.*

Corresponding author: Wenqi Song, soongwenqi@163.com; You Yu, yuyou@shanghaitech.edu.cn

Sci. Adv. **11**, eadw3725 (2025)
DOI: 10.1126/sciadv.adw3725

This PDF file includes:

Supplementary Text
Figs. S1 to S38
Table S1
References

Supplementary Text

Adaptive machine learning for gestures classification with FeatureLoss

Real-time signals from the new user were transformed using this matrix. After the LCN process, the parameters in the early layers of the pre-trained network were frozen, and new user data was mixed with standard data at ratios of 1:5 and 1:1 to prevent overfitting. This mixed dataset was then fed into the pre-trained network to fine-tune the parameters of the final layers.

LCN was designed to enhance the trial adaptability, addressing discrepancies in both electrode position and the variability of the sEMG itself across different individuals or trials. LCN applied a linear combination to recombine the channels into a new 8-channel signal. This transformation was performed using a trial specific transformation matrix, which was generated iteratively to align the new user's signals with the standard sEMG used in pre-training. We introduced the loss function (FeatureLoss) to compute the disparity between motion occurrence segment extracted from standard sEMG signals and new sEMG signals. For standard sEMG signals $X_{std}(t)$ and new sEMG signals $X_{new}(t)$, gesture-specific occurrence segments are extracted during motion events. For each gesture g, we extracted segments $X_{std}^{(g,i)}$ and $X_{new}^{(g,i)}$ by peak detection. Once the segments were identified, we randomly selected pairs of segments corresponding to the same gesture from the new user and standard dataset, ensuring that both segments were from the same gesture g. The FeatureLoss loss function was designed to quantify the disparity between the paired segments, focusing on the maximum value and standard deviation of the signal. These two features were selected because maximum value Indicates muscle activity intensity and strength of contractions, and standard deviation reflects muscle activity stability and fatigue. These features effectively capture movement characteristics and individual differences, significantly enhancing signal alignment in the LCN process. The FeatureLoss for each positive pair of segments $X_{std}^{(g,i)}$ and $X_{new}^{(g,j)}$. is given by the dual loss function:

$$\mathcal{L}_{\text{FeatureLoss}} = \frac{1}{N}\sum_{i=1}^{N}\left(\alpha \cdot \left|max(X_{\text{new}}^{(g,i)}) - max(X_{\text{std}}^{(g,j)})\right| + \beta \cdot \left|\sigma(X_{\text{new}}^{(g,i)}) - \sigma(X_{\text{std}}^{(g,j)})\right|\right)$$

Where α and β are hyperparameters that control the relative importance of the two components.

the transformation matrix W is used to map the new sEMG signal segments closer to the standard signal segments. This transformation is iteratively optimized by minimizing the FeatureLoss loss function using the backpropagation algorithm. The objective is to update W such that the transformed new sEMG segments $\hat{X}_{\text{new}}^{(g,i)} = W \cdot X_{\text{new}}^{(g,i)}$ are as close as possible to the standard segments $X_{\text{std}}^{(g,i)}$. After LCN processing, a new user's 8-channel signal could be transformed into a new 8-channel form, closely resembling the standard sEMG gestures, thereby improving the accuracy of gesture classification.

Methodology for Uniaxial Tensile Testing and Data Analysis

1. Specimen Preparation

Dumbbell-shaped specimens were fabricated in accordance with the ASTM D412 standard. Materials such as Ecoflex and Polydimethylsiloxane (PDMS) were cast into 3D-printed molds to create the specimens. After curing at room temperature, the specimens were carefully demolded.

Uniaxial tensile tests were performed on an Instron 68S-C universal testing machine (Instron, Norwood, MA), equipped with a 100 N load cell. Before initiating each test, the measured geometric parameters of the specimen were input into the system's software. During the tests, specimens were subjected to displacement-controlled loading at a constant crosshead speed of 100 mm/min until fracture. To evaluate the reliability and repeatability of the test protocol, a total of three independent specimens were tested under identical conditions.

2. Data Processing and Statistical Analysis

Data processing commenced with the true stress and true strain curves, which were directly generated by the testing software using the pre-input specimen geometry. These raw curves, formed the basis for statistical analysis to assess measurement uncertainty and to generate a mean curve with its corresponding confidence interval for constitutive model fitting.

A Type A evaluation of measurement uncertainty was conducted to statistically analyze the data from the three replicate tests. At each point of true strain, the following calculations were performed:

2.1 Calculation of the Arithmetic Mean ($\bar{y}$):

The mean of the true stress values (y_1, y_2, y_3) from the three tests was calculated. These mean values constitute the final "mean curve."

$$\bar{y} = \frac{1}{n}\sum_{i=1}^{n} y_i$$

2.2 Calculation of the Corrected Sample Standard Deviation (s):

This value quantifies the dispersion of the measurement data. The use of the n-1 denominator provides an unbiased estimate of the population standard deviation.

$$s = \sqrt{\frac{1}{n-1}\sum_{i=1}^{n}(y_i - \bar{y})^2}$$

2.3 Calculation of the Standard Deviation of the Mean ($s_{\bar{x}}$):

This value, also known as the standard error, represents the statistical dispersion of the sample mean and is the expression of the statistical dispersion.

$$s_{\bar{x}} = \frac{s}{\sqrt{n}}$$

2.4 Calculation of the 95% Confidence Interval (CI):

The 95% CI for the mean curve was computed to define a confidence range for the data, which is essential for robust model fitting.

$$\text{CI} = \bar{y} \pm t \cdot s_{\bar{x}}$$

where t is the critical value from the t-distribution for a 95% confidence level and $n-1$ degrees of freedom (for n=3).

Finally, the resulting mean stress-strain curve and its 95% confidence interval were used for fitting hyperelastic constitutive models in simulation software to characterize the mechanical behavior of the silicone material.

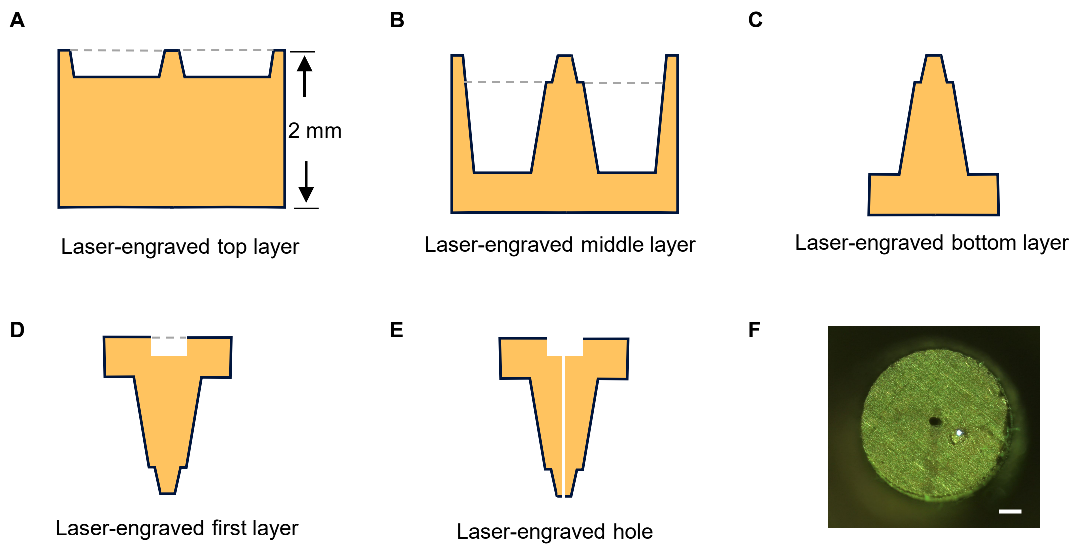

Fig. S1. Schematic and fabrication procedure of printing nozzle. (**A** to **E**) Fabrication processes of a high-resolution nozzle with laser engraved. (**F**) Microscopic image for laser manufactured copper nozzle. Scale bar, 150 μm.

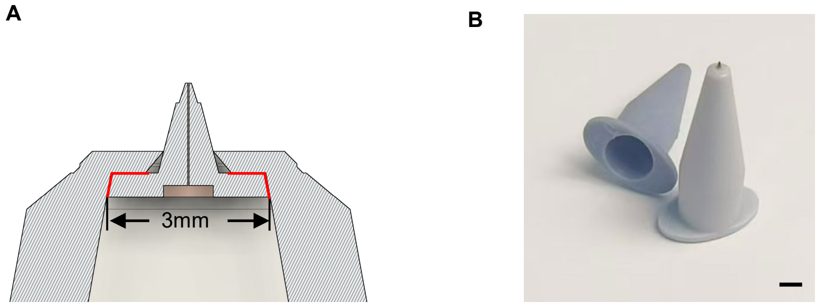

Fig. S2. Schematic and image of printing syringe. (**A**) Cross section of syringe with the nozzle, red line indicating the sticky interface. (**B**) Photograph of prepared syringes with nozzles. Scale bar, 3 mm.

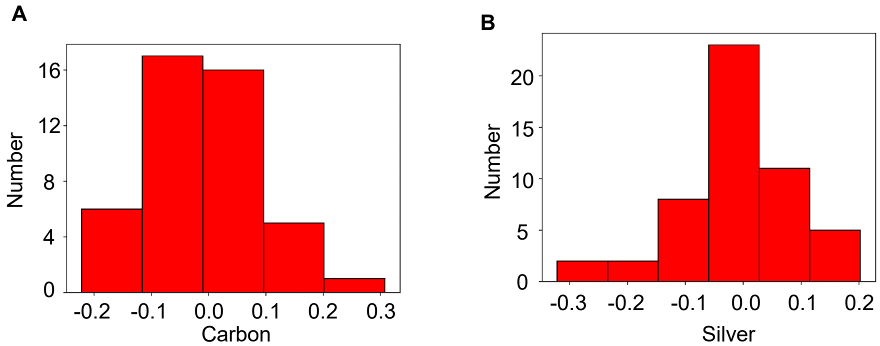

Fig. S3. Characterization of the printed electrodes. Distributions of normalized differences from the mean value printed by carbon ink (**A**) and silver ink (**B**).

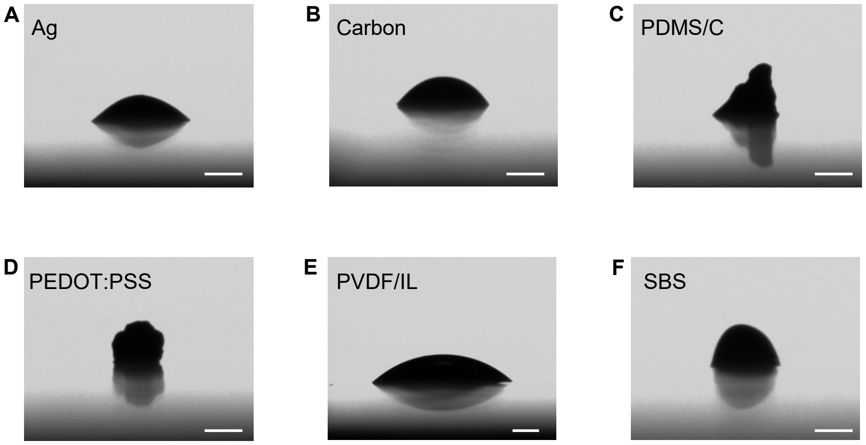

Fig. S4. Wettability characterization of the customized inks. Photographs of Ag ink (**A**) and carbon ink (**B**), PDMS/C ink (**C**), PEDOT:PSS ink(**D**), PVDF/IL ink (**E**) and SBS ink (**F**). Scale bars, 1 mm.

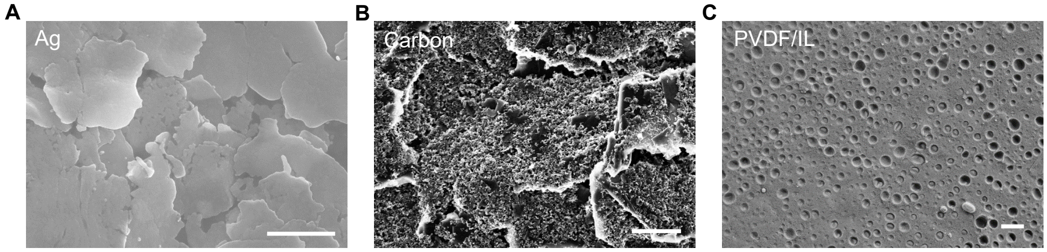

Fig. S5. Characterization of the printing inks. SEM images of pattern surface printed by Ag ink (**A**) and carbon ink (**B**), PVDF/IL ink (**C**). Scale bars, 2 μm.

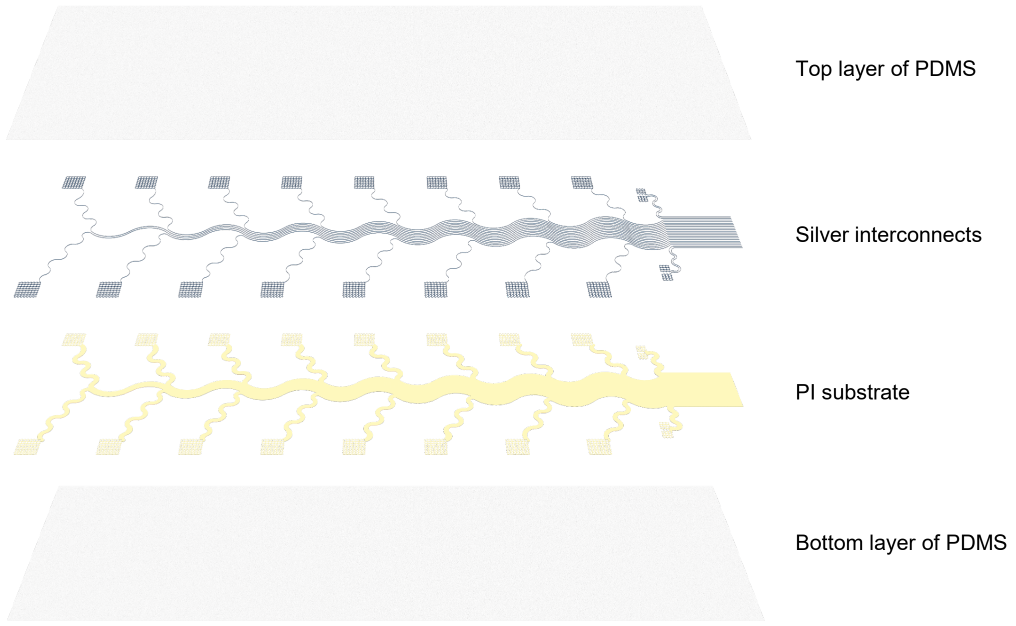

Fig. S6. Exploded view of e-skin with sEMG electrode and stimulation electrode. Schematic of the soft e-skin consist of silver interconnect layer, PI substrate, and PDMS encapsulation layers.

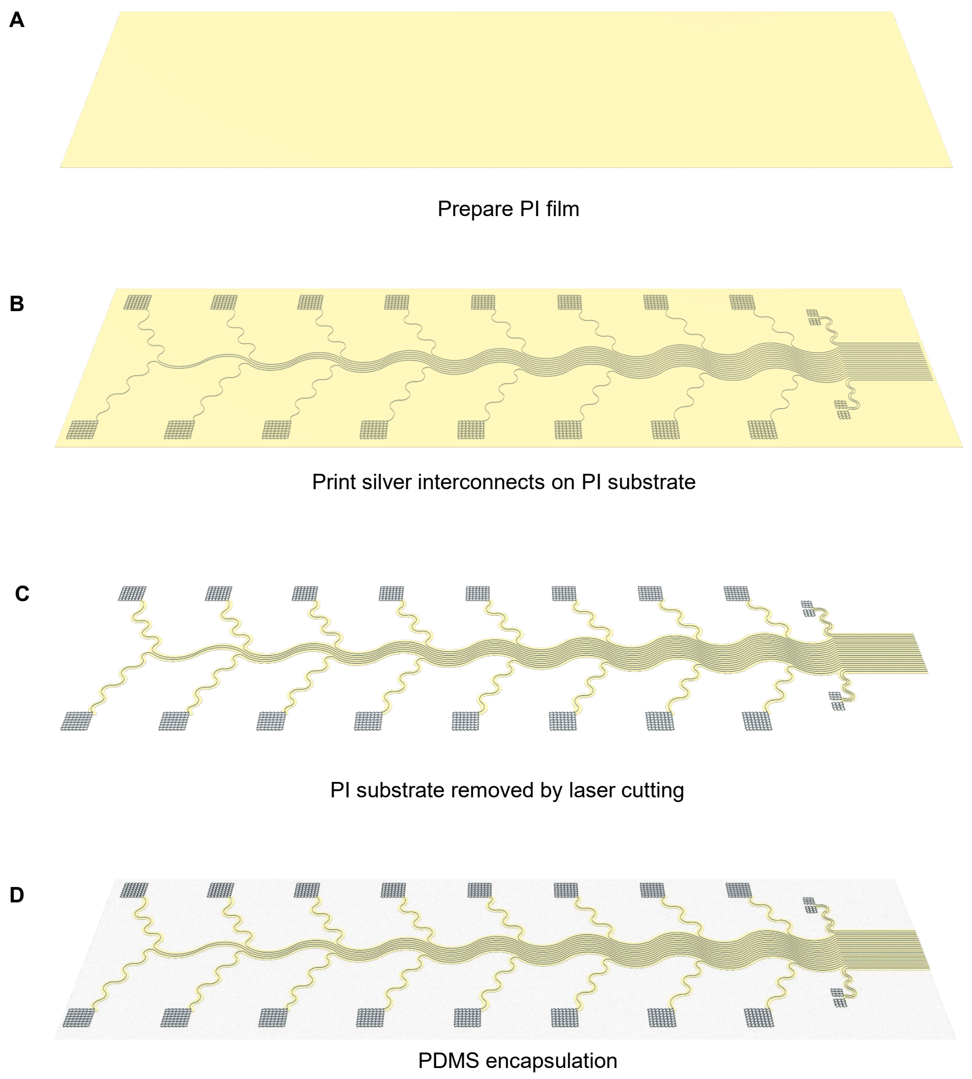

Fig. S7. Fabrication processes of e-skin with printing and laser cutting. (**A**) PI substrate cleaning. (**B**) Silver interconnects were printed on PI substrate. (**C**) PI substrate was removed by laser cutting. (**D**) PDMS encapsulation on the top layer.

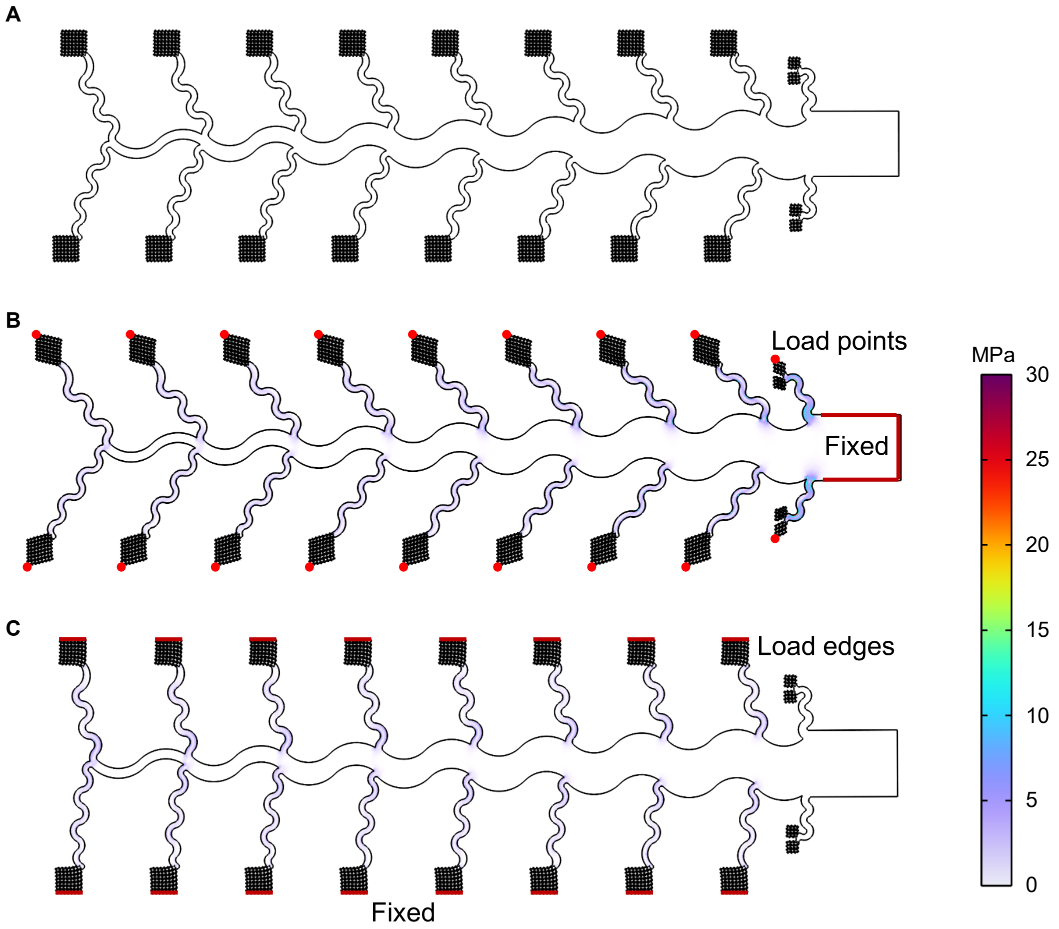

Fig. S8. Simulation of the sEMG electrode. Mechanical simulation of e-skin of origin state (**A**), longitudinal direction load (**B**) and transverse direction load (**C**).

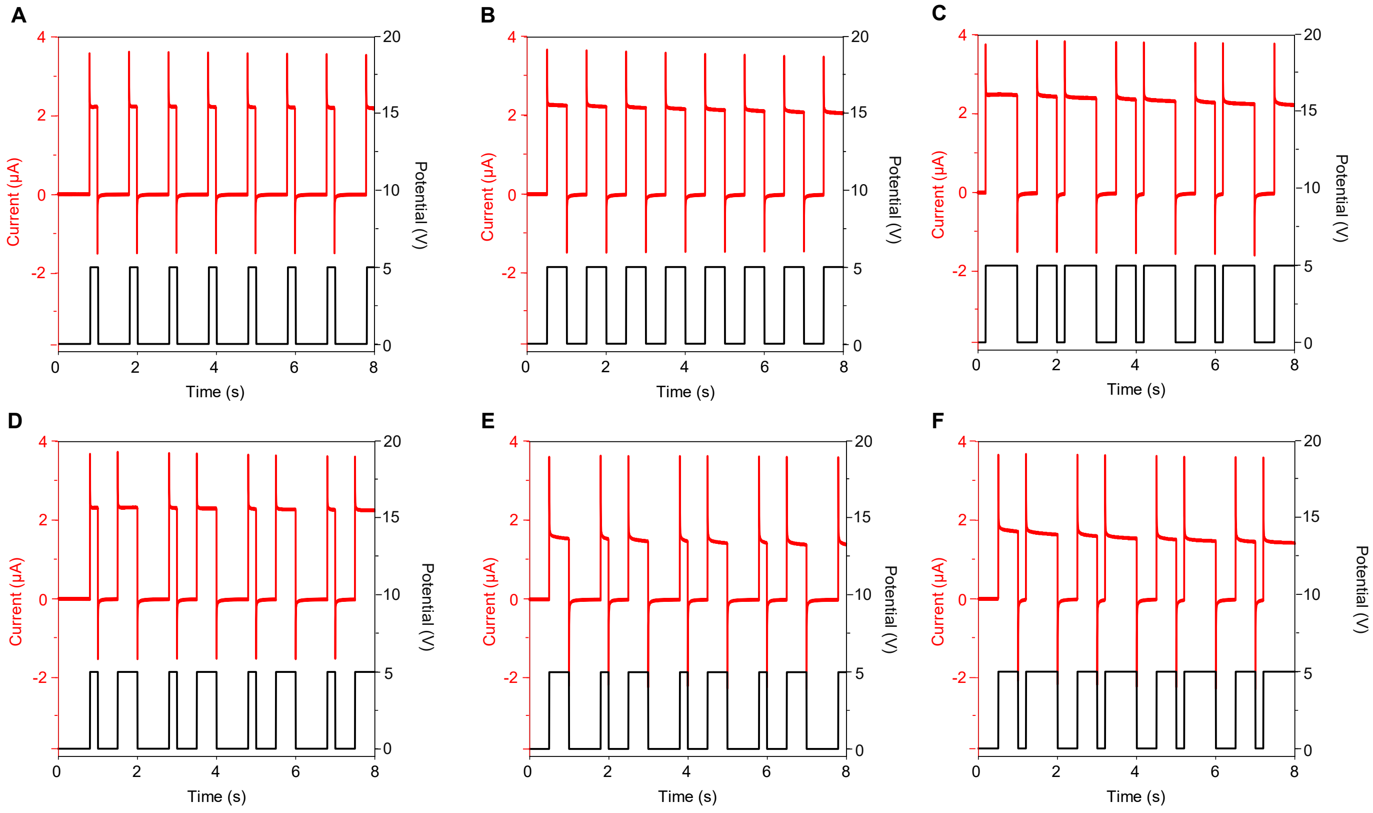

Fig. S9. Characterization of the stimulation electrodes. Current feedbacks on the subject's arm with programmed applied potentials (**A** to **F**).

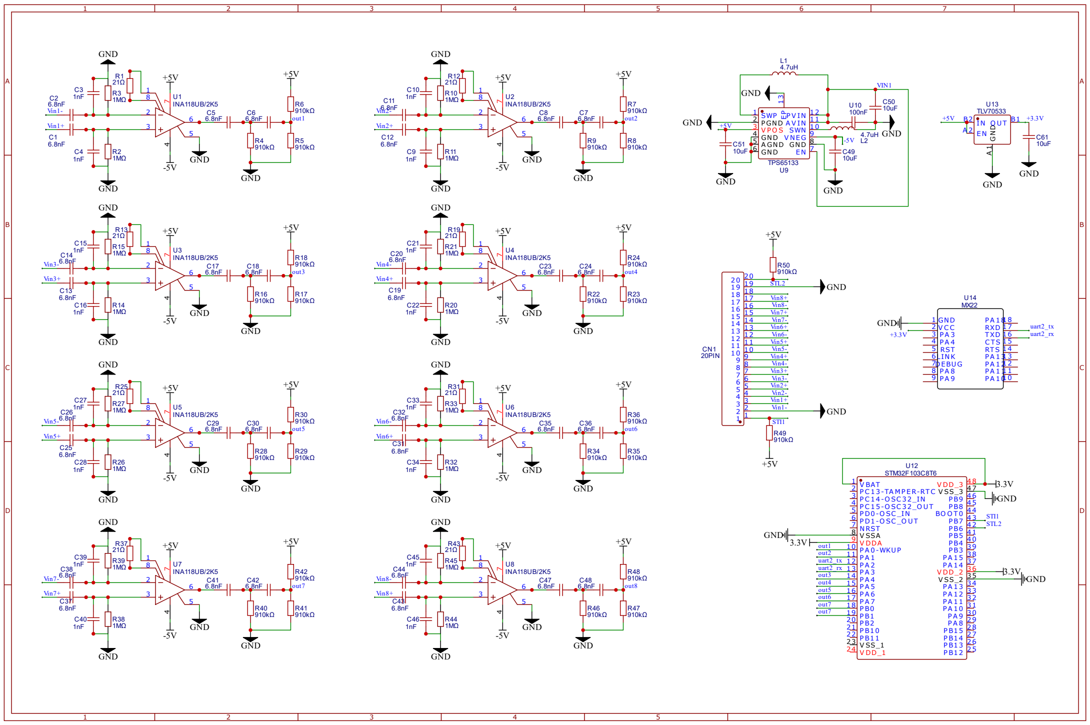

Fig. S10. Schematic illustration of soft circuit board for sEMG signal acquiring and transmission.

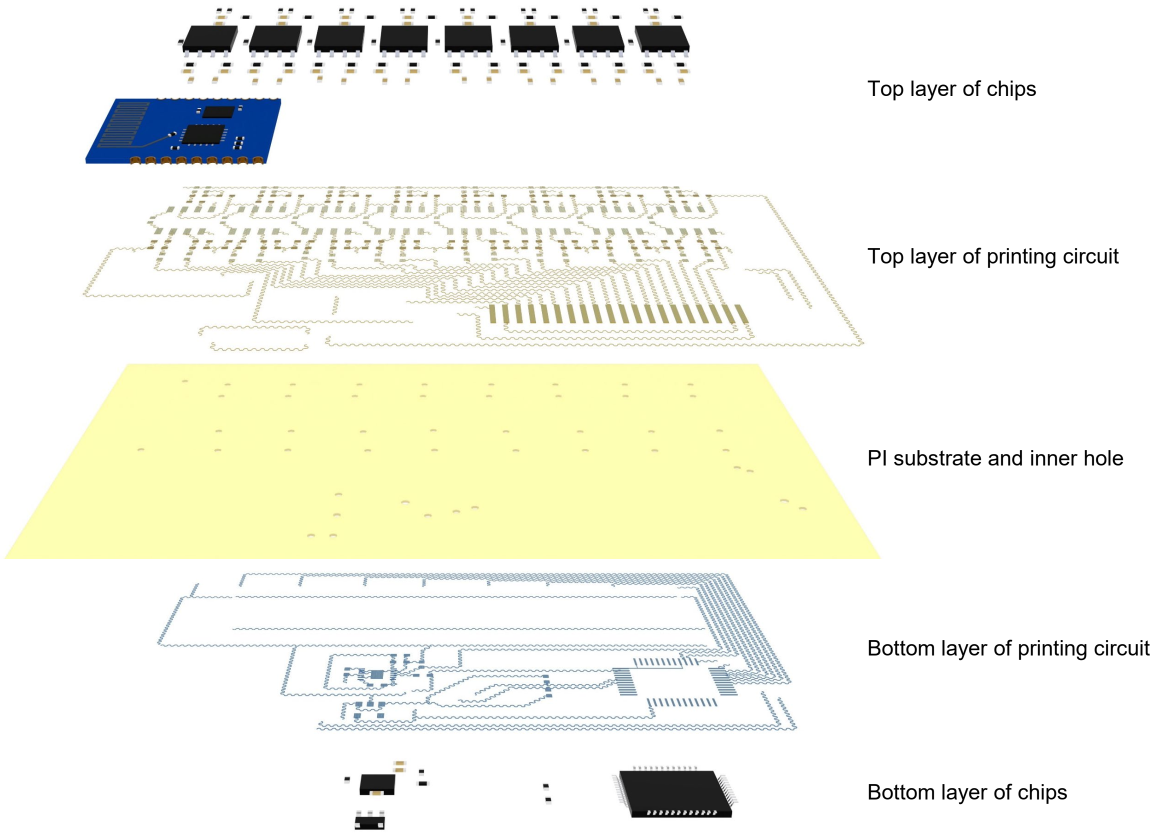

Fig. S11. Schematic of soft circuit board component of different layers.

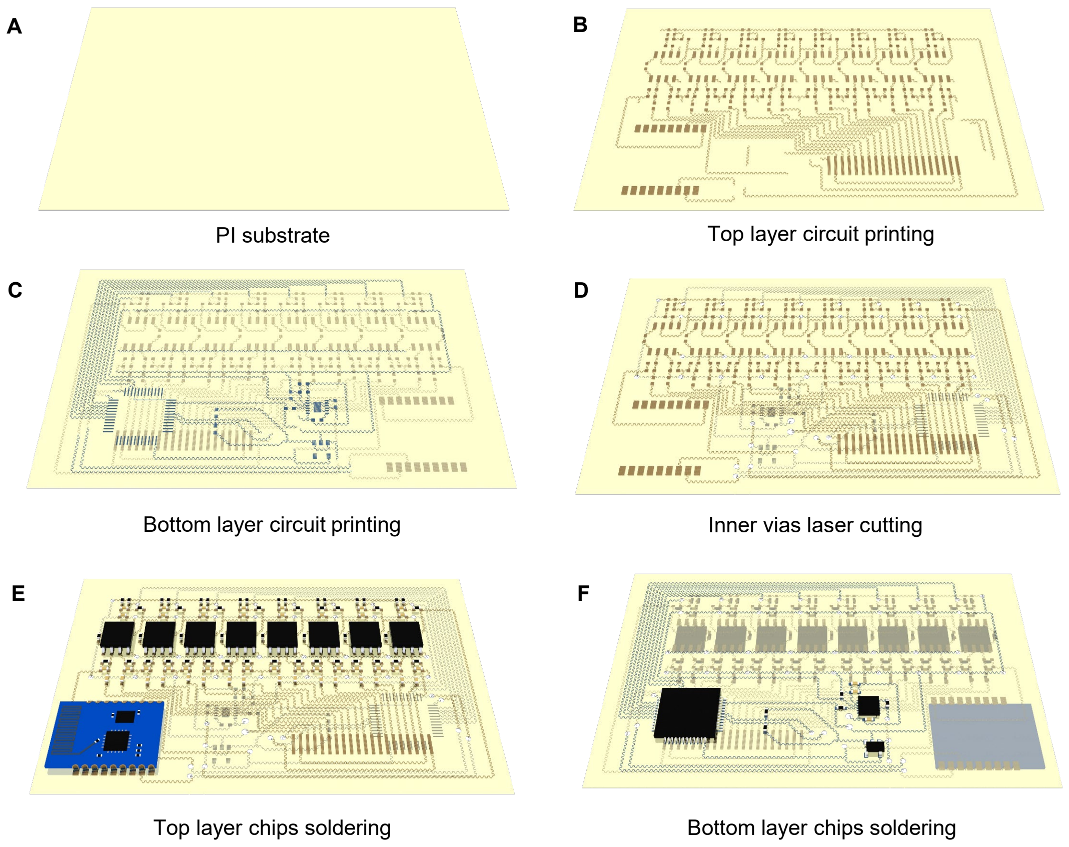

Fig. S12. Schematic of soft circuit board fabrication processes. (**A**) PI substrate cleaning. (**B**) Silver interconnects printed on top layer. (**C**) Silver interconnects printed on bottom layer. (**D**) Inner holes were generated by laser cutting. (**E**) Electric components were soldered on the top layer. (**F**) Electric components were soldered on the bottom layer.

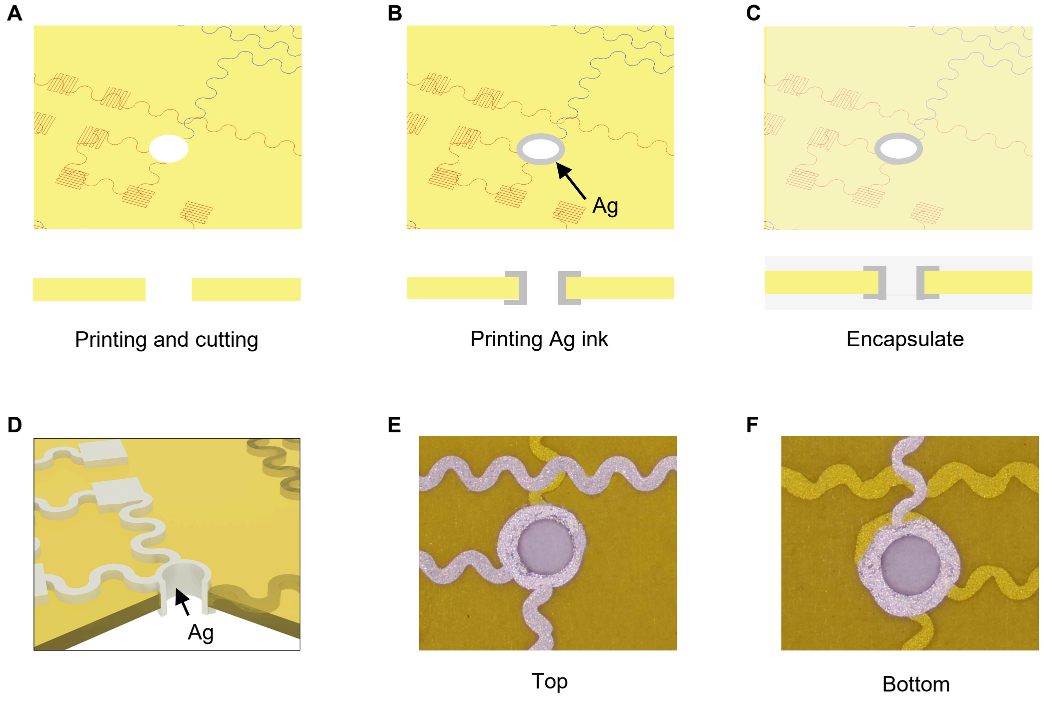

Fig. S13. Illustration of inner vias fabrication. (**A**) to (**C**) schematic of vias conductive fabrication with cutting a hole on a printed PI, printing enough amount Ag ink around the hole, and encapsulate by PDMS. (**D**) 3D structure of vias by Ag connect top and bottom side. (**E**) Optical image of vias top side. (**F**) Optical image of vias bottom side. Scale bars,

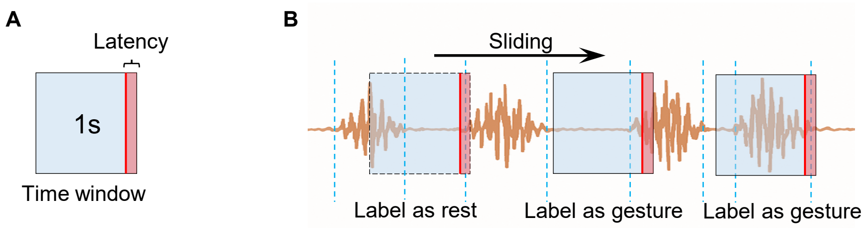

Fig. S14. Dataset preparation using a designed time window and latency labeling method. **(A)** A 1 s time window within a designed latency for cropping raw data. (**B**) Detailed dataset preparation processes. The time window was sliding across on sEMG data. Blue dash line, boundary of a gesture sEMG signal.

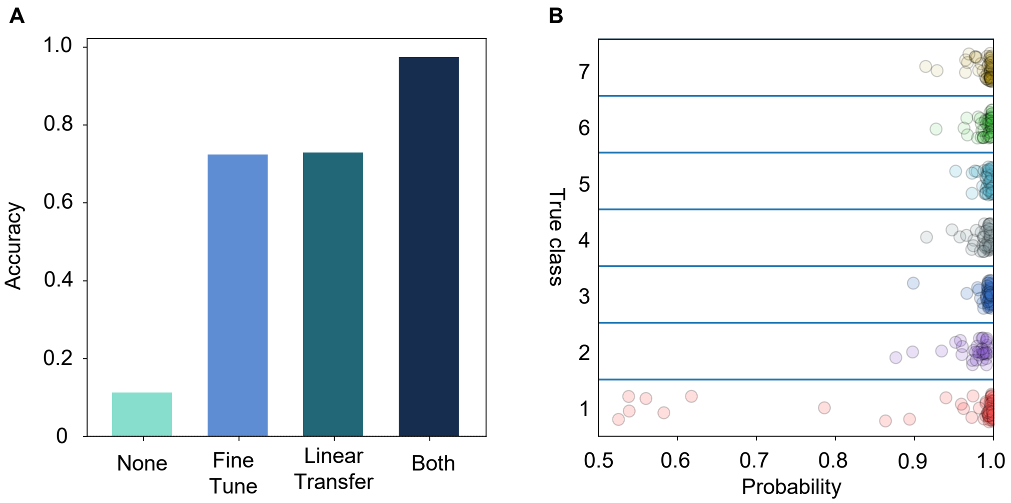

Fig. S15. Classify results with 6 kinds gestures . (**A**) Compare of classification accuracy among LMN, FTP, and both models. (**B**) Relationship of training epoch and test accuracy with different time latency. Inset, relationship between the time latency and accuracy.

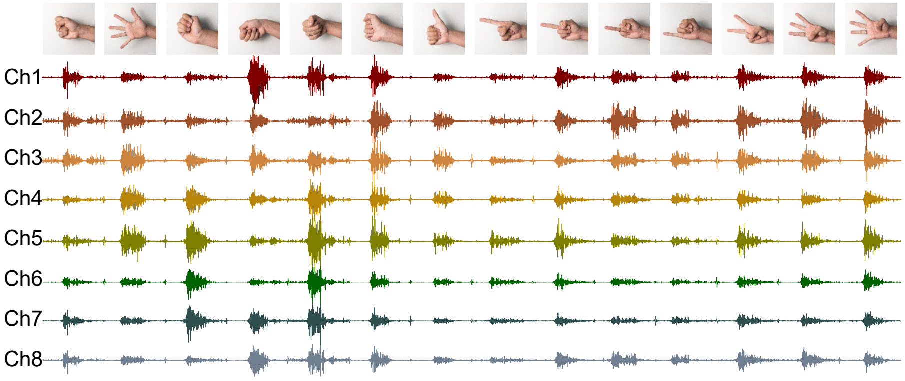

Fig. S16. sEMG signal visualization of 14 kinds of gestures. Inset, gestures from left to right are fist, release, up, down, left, right, thumb finger, index finger, middle finger, ring finger, little finger, and finger gestures of two, three and four, respectively.

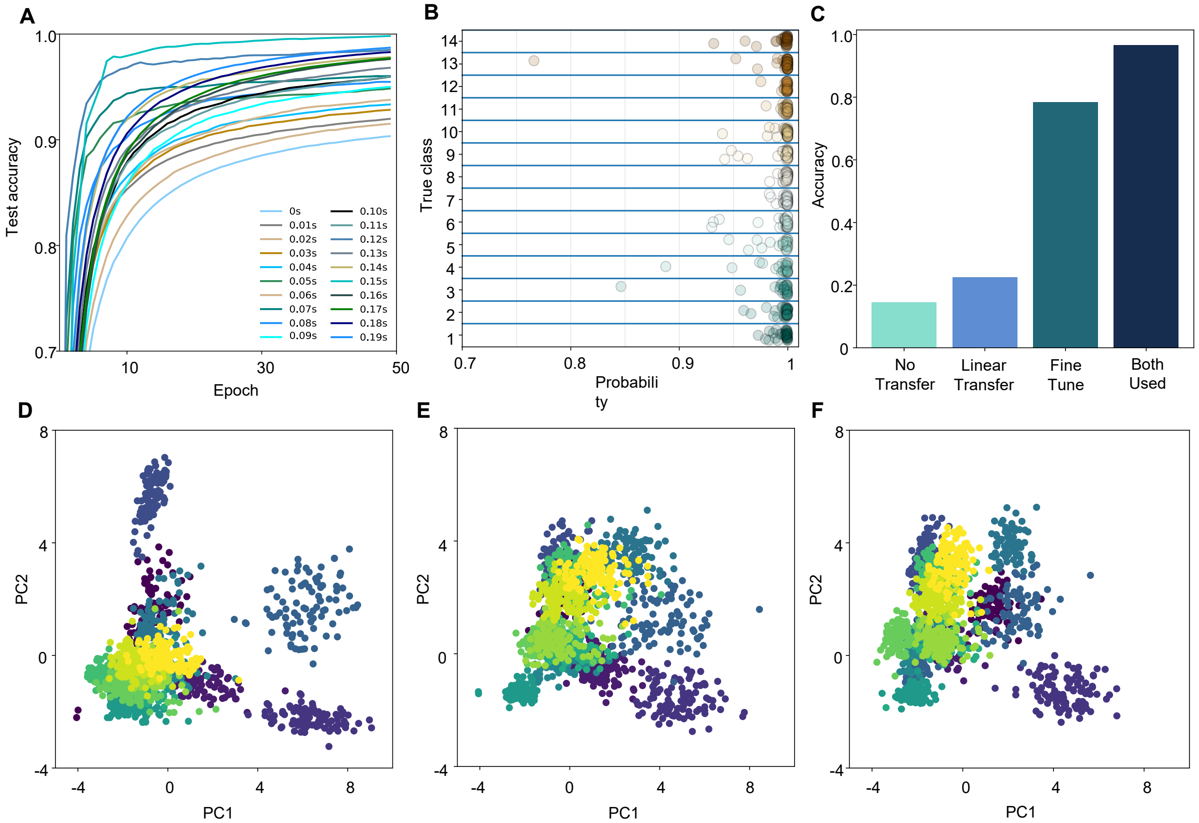

Fig. S17. Gesture classification obtained with adaptive machine learning with 14 gestures. (**A**) Relationship of training epoch and test accuracy with different time latency in 6 hand gestures dataset. (**B**) Confidence probability of the individual adaptable machine learning algorism classification result of 14 hand gestures dataset. (**C**) Compare of classification accuracy among LMN, FTP, and both models in 14 hand gestures dataset. (**D**) Raw data distribution of all the 13 kinds of gestures. Target data distribution (**E**) from a human subject and distribution after LMN process (**F**).

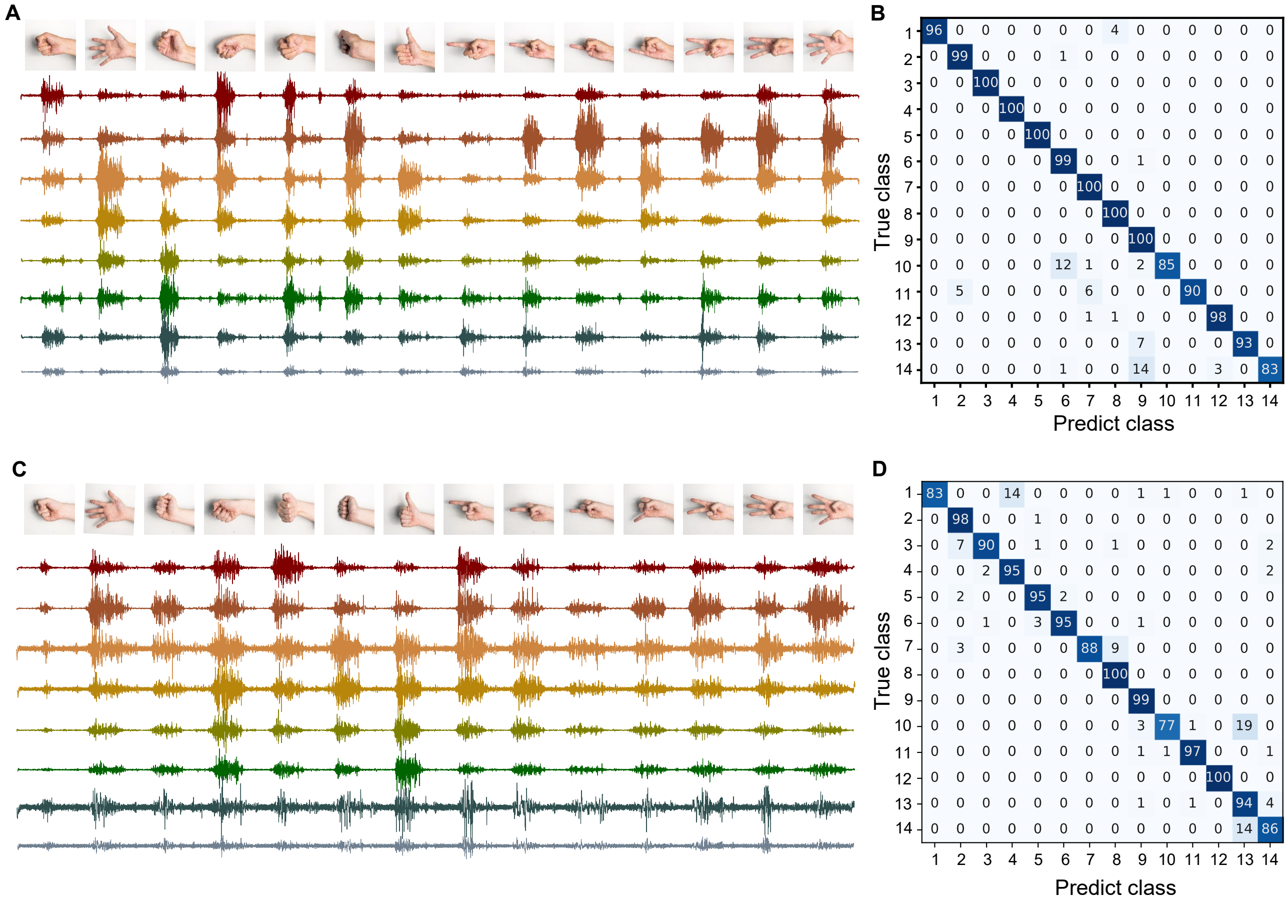

Fig. S18. Evaluation of the sEMG electrode with adaptive machine learning. sEMG signals (**A** and **C**) and classification matrix (**B** and **D**) of 8 channels from 14 hand gestures with the adaptive machine learning model from different subjects, respectively.

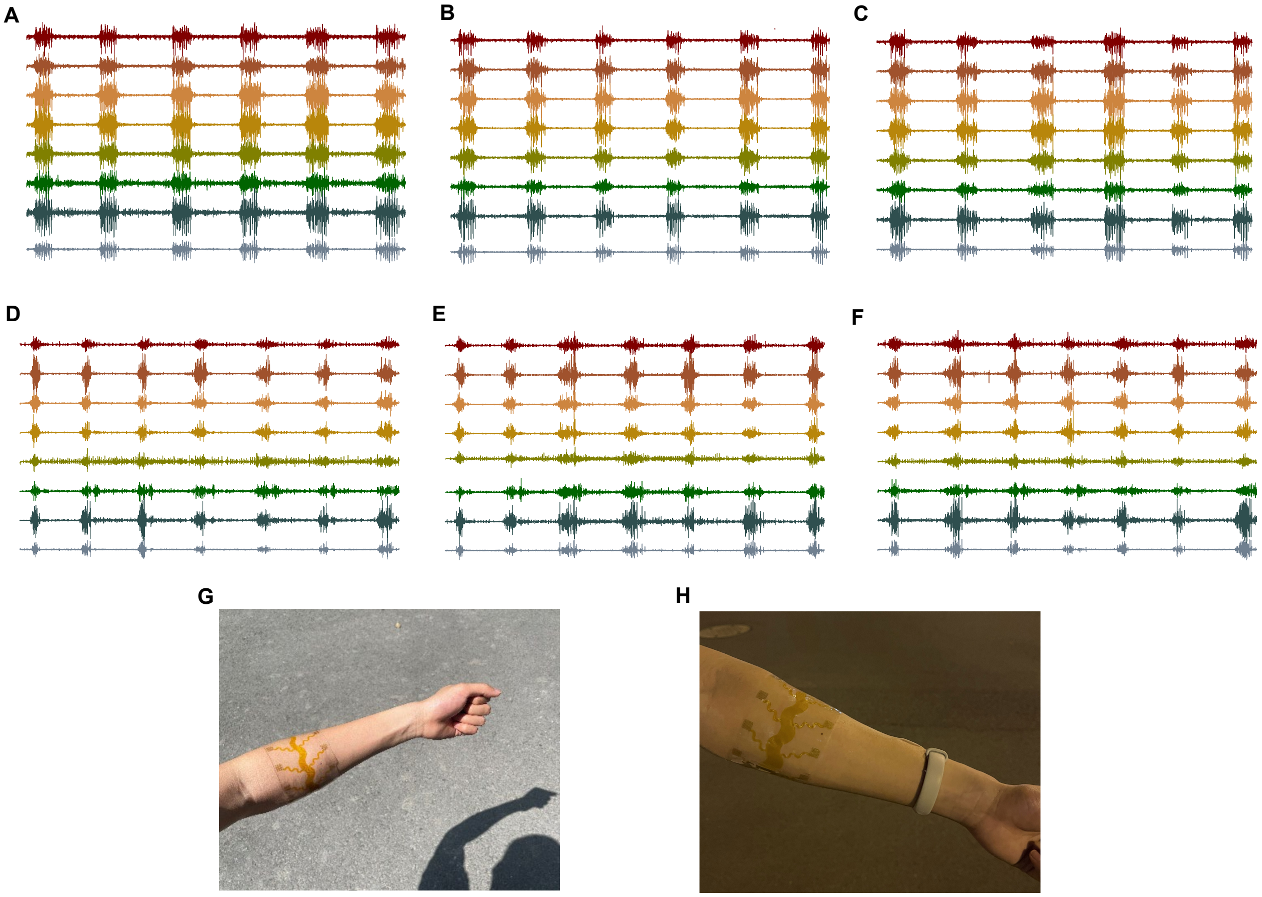

Fig. S19. Evaluation of the sEMG electrode before and after exercises. (**A** to **C**) sEMG signals of fist gesture from a subject before (A) and after (B) exercises in the daylight and at night (C). (**D** and **F**) sEMG signals of fist gesture from another subject before (D) and after (E) exercises in the daylight and at night (F). (**G** and **H**) Photographs of a subject wearing the sEMG electrodes in the daylight (G) and at night (H).

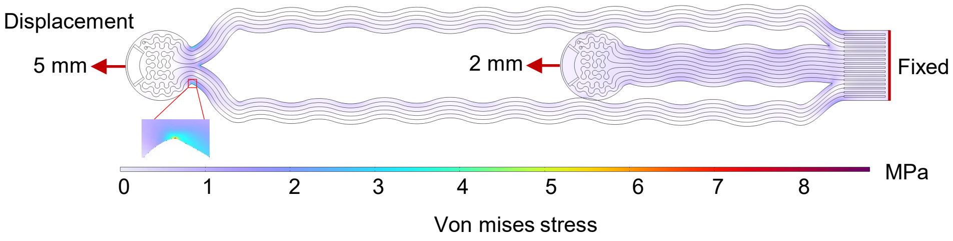

Fig. S20. Mechanical simulation of sensor unit under a displacement with boundary conditions.

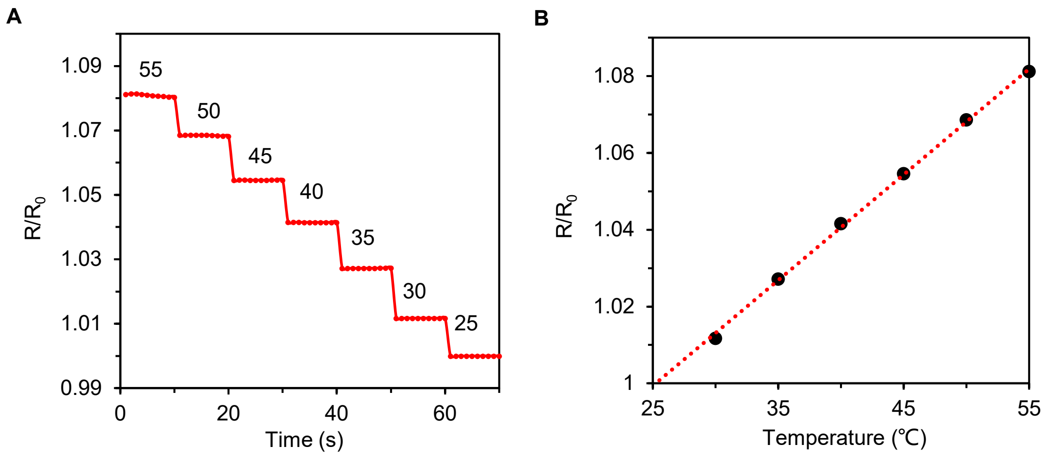

Fig. S21. Cooling process characterization of temperature sensor. (**A**) Relative resistance change with decrease temperature and relative average resistance along different temperature with linear fit result (**B**).

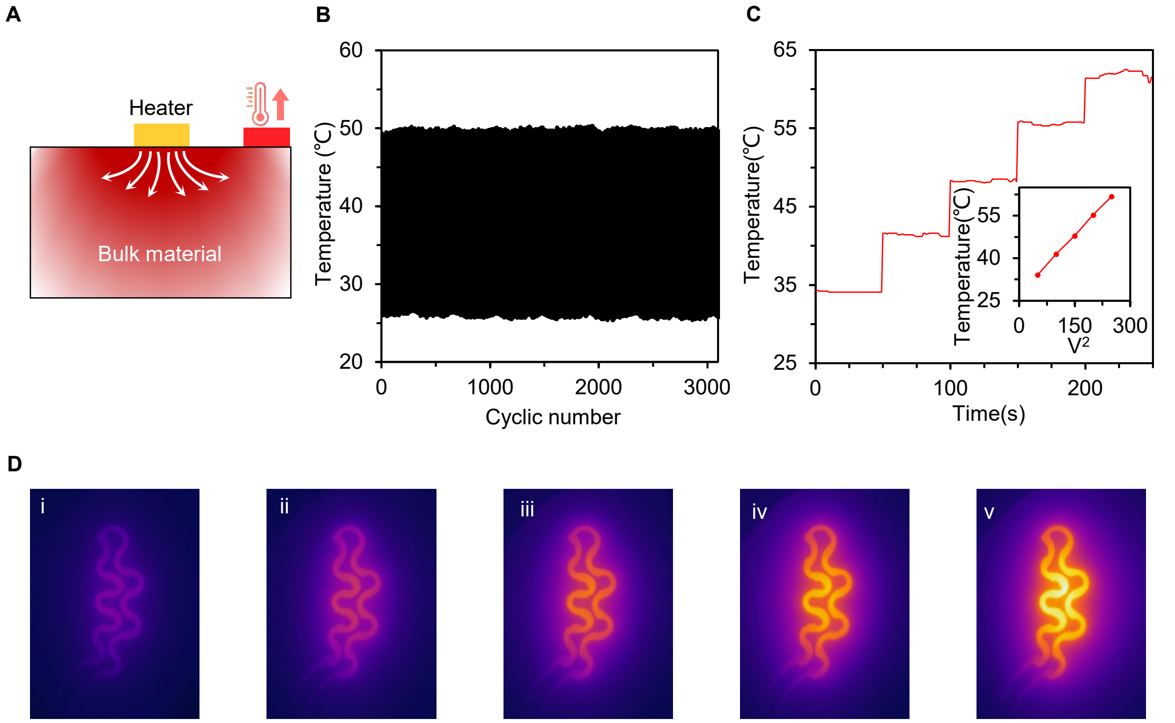

Fig. S22. Schematic and characterization of the thermal conductivity sensors. (A) Schematic of thermal conductivity test principle. (**B**) Repeatability test of the heater performance (**C**) Response and the calibration plots (inset) of a heater under varied voltage applied. (**D**) Infrared images of the heater under varied potential applied.

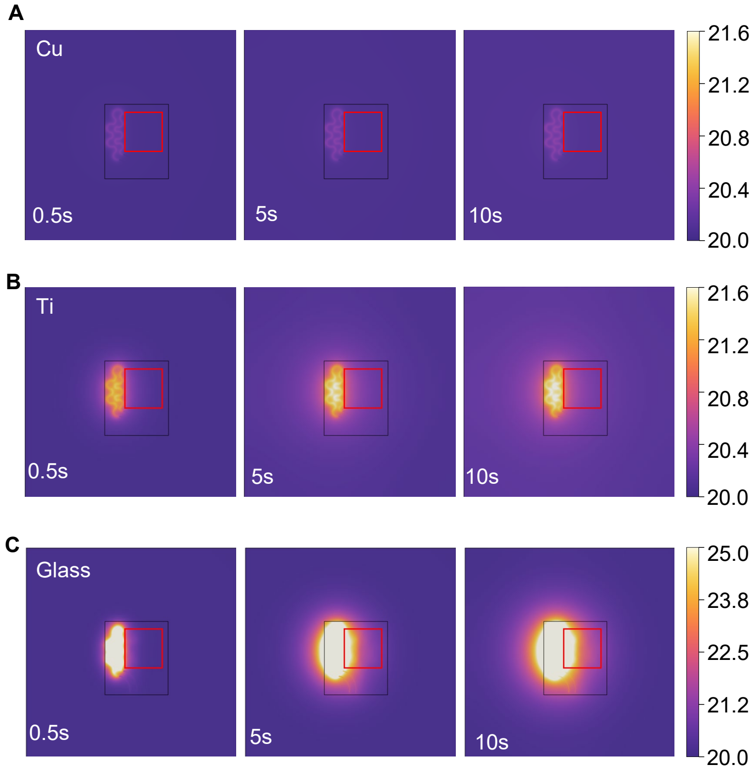

Fig. S23. Simulation of the thermal conductivity tests on different materials. Simulated results of thermal conductivity test with heaters and temperature sensors at copper (**A**), titanium (**B**), and glass (**C**), respectively.

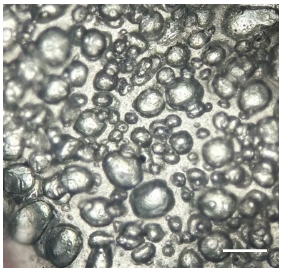

Fig. S24. Image of bubble enabled PDMS/C layer. Scale bar, 100 µm.

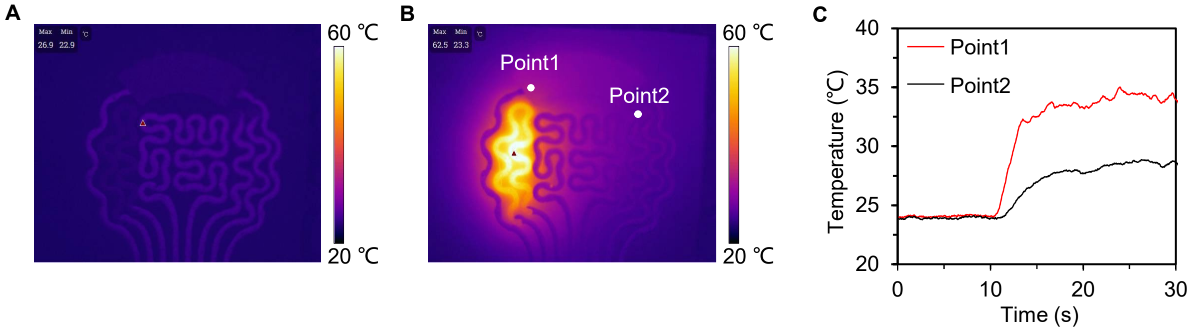

Fig. S25. Temperature change on pressure by heater working. (**A**) Thermal image of a multimodal sensor at room temperature. (**B**) Thermal image of a multimodal sensor under heater working with max power. (**C**) Temperature change of sampling point1 and point2. Red triangle indicate highest point in the picture.

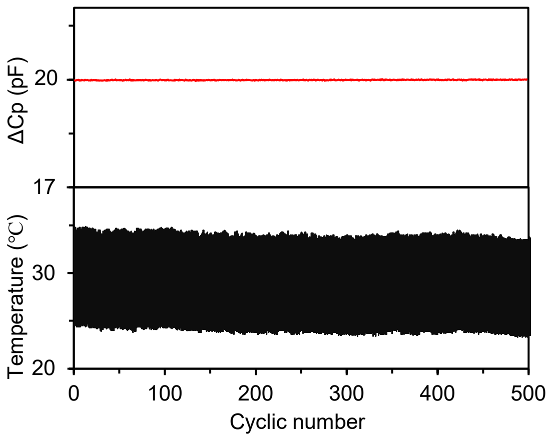

Fig. S26. Stability of pressure sensor with cyclic temperature range from 25 to 35 °C.

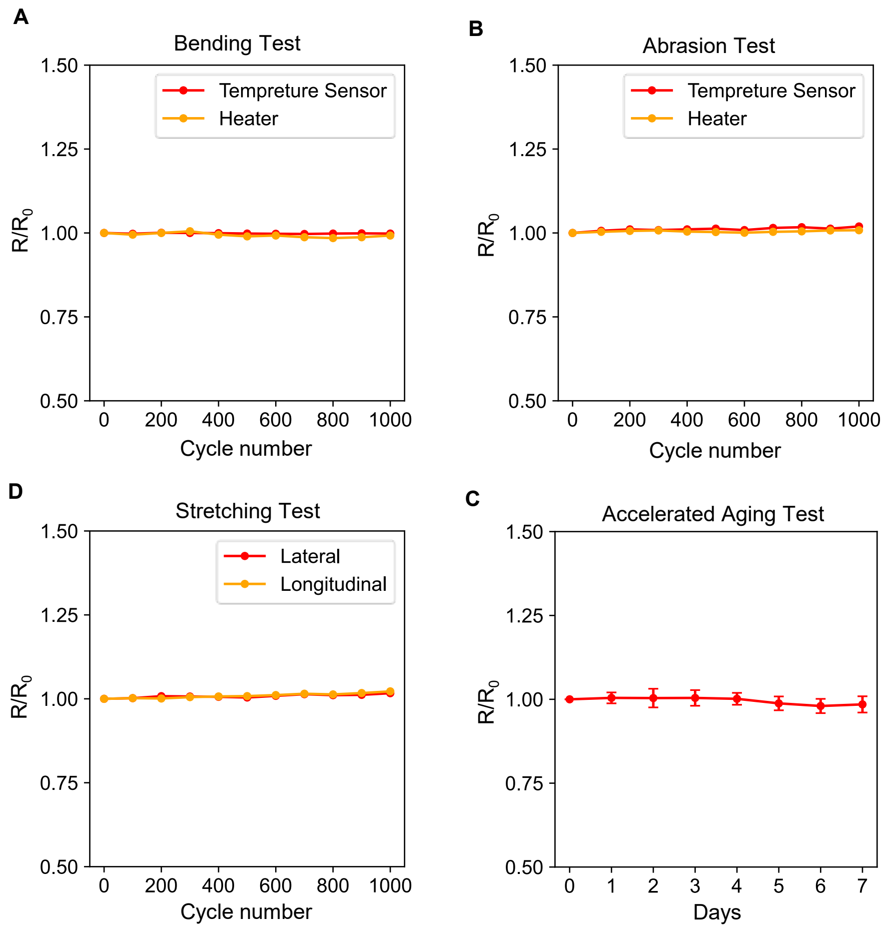

Fig. S27. Mechanical and environmental stability evaluation of the printed e-skin and integrated sensors. (**A**) Resistance changes of temperature sensor and heater during repeated bending on a finger. (**B**) Resistance changes under repeated friction using a rough plane with 0.1 mm protrusions and 3.9 kPa contact pressure. (**C**) Resistance response of the e-skin under 6% lateral and longitudinal strain. (**D**) Accelerated aging test (85°C/85% RH): long-term resistance stability of the Temperature Sensor over 168 hours.

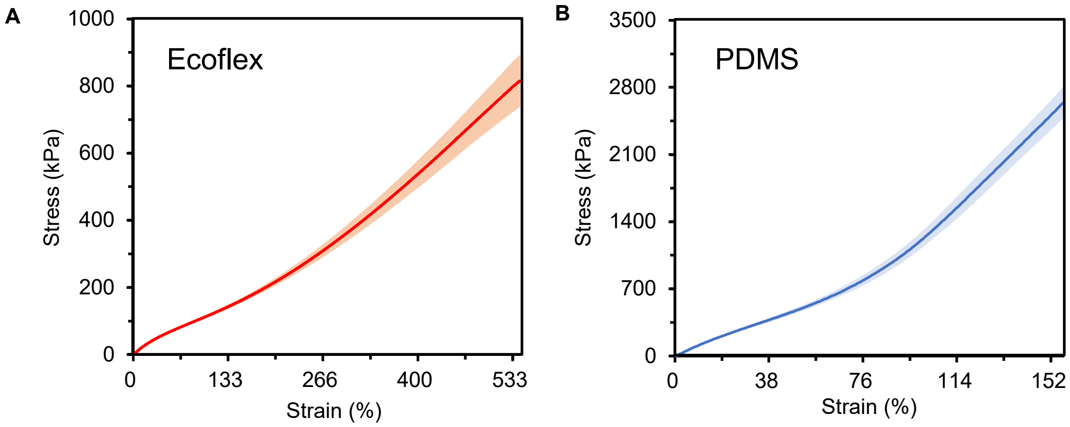

Fig. S28. Experimental uniaxial tensile Stress–Strain pull to failure responses of Ecoflex (A) and PDMS (B) with 95% confidence bands from 3 repeated tests.

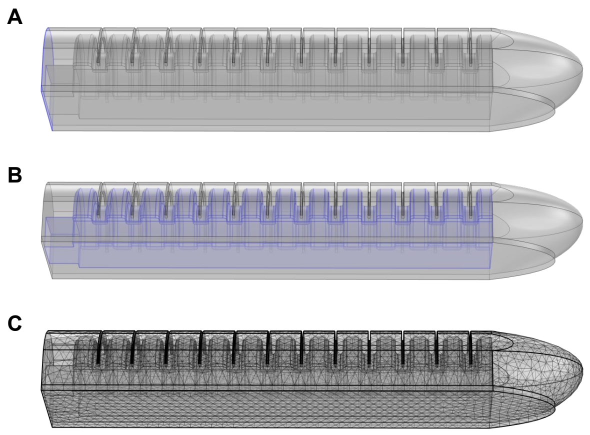

Fig. S29. Detail of robotic finger in simulation. Fixed constraint(**A**), applied uniform pressure (**B**) and free tetrahedral mesh (**C**).

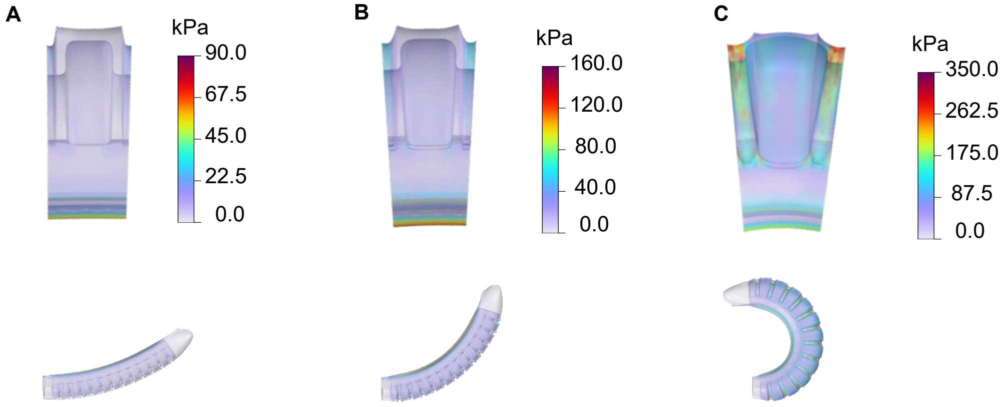

Fig. S30. Simulation of the soft robotic fingers. Simulated results of robotic fingers with different angles of 30° (**A**), 70° (**B**), 180° (**C**). Angle defined by the central angle of a fitting circle from bending shape.

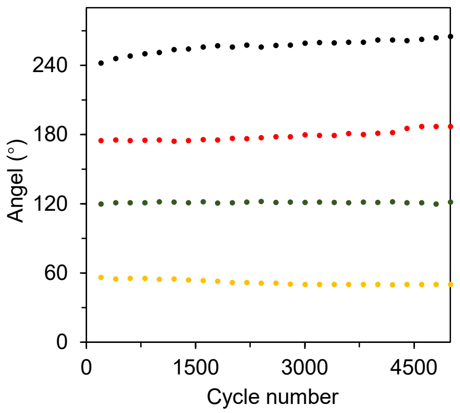

Fig. S31. Repeatability test of soft robot finger at different bending angles.

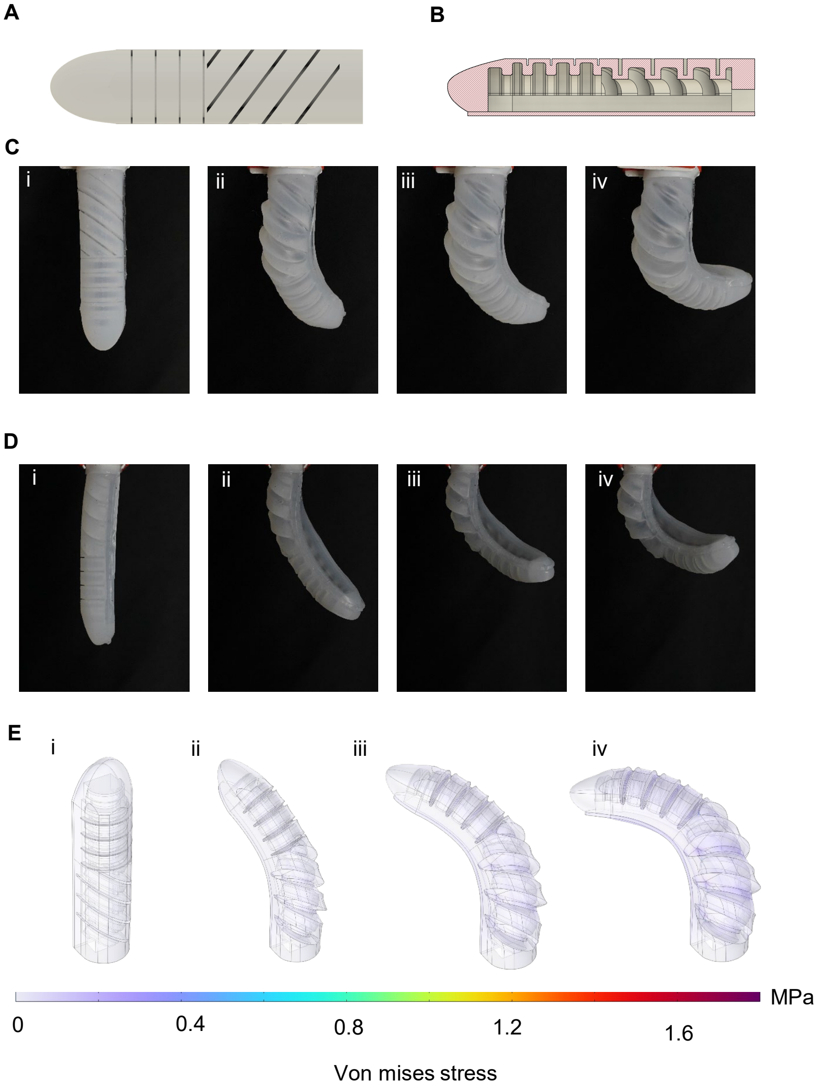

Fig. S32. Design and evaluation of thumb finger. Top view (**A**) and cross section view (**B**) of thumb finger design. Photographs of front view (**C**) and side view (**D**) along with the pressure increasing. (**E**) Mechanical simulation result of thumb finger under different air pressure.

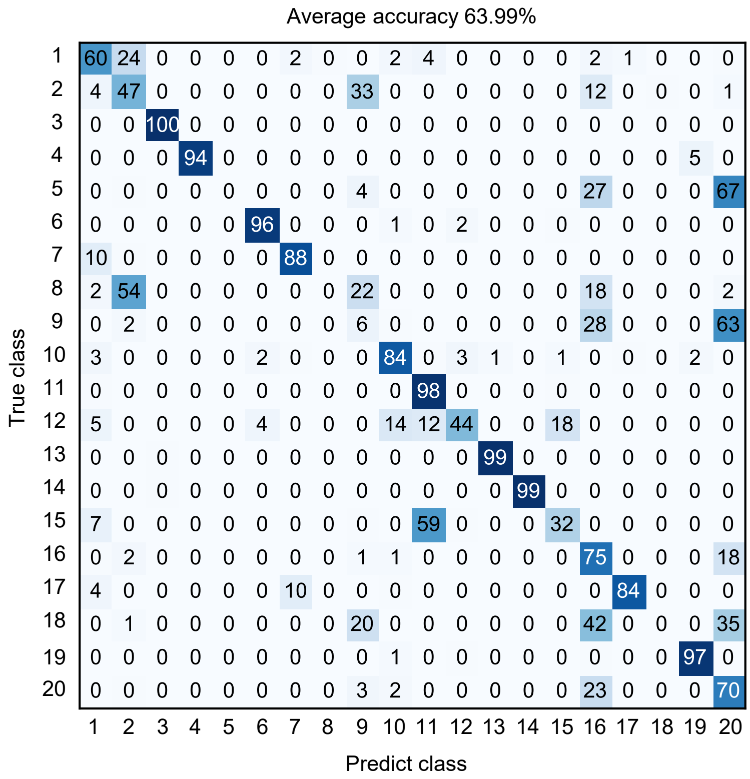

Fig. S33. Confusion matrix of classification result of the 20 objects with thermal conductance data.

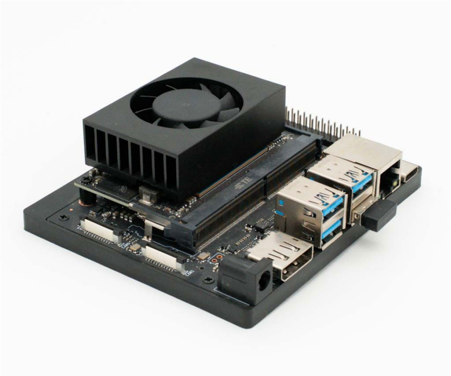

Fig. S34. Photograph of the hardware with CNN for material recognition.

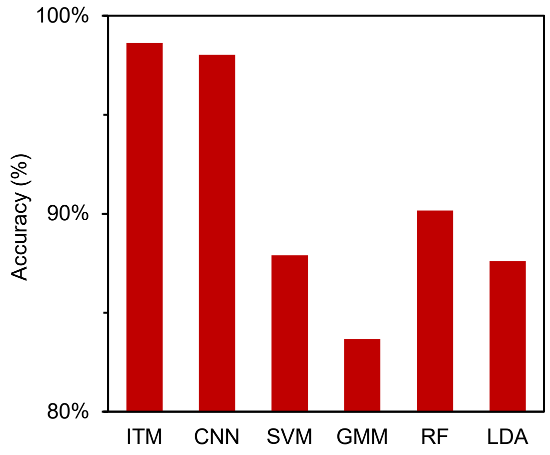

Fig. S35. Comparation between different models for object recognition. Support Vector Machine (SVM), Gaussian Mixture Model (GMM), Random Forest (RF), and Linear Discriminant Analysis (LDA).

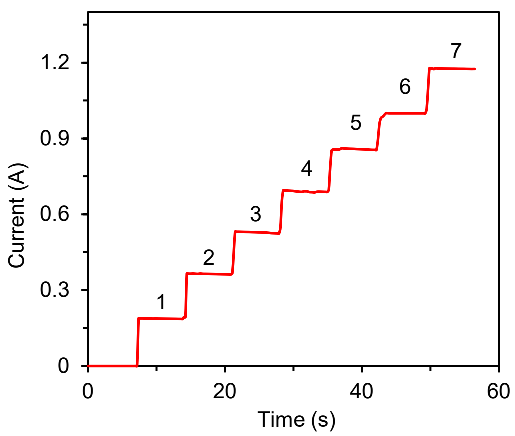

Fig. S36. Current recorded at 12 V with different working state. (1 to 5) The inflation valves to each soft robotic finger were sequentially turned on (6) The air pump was turned on. (7) the deflation valve was turned on.

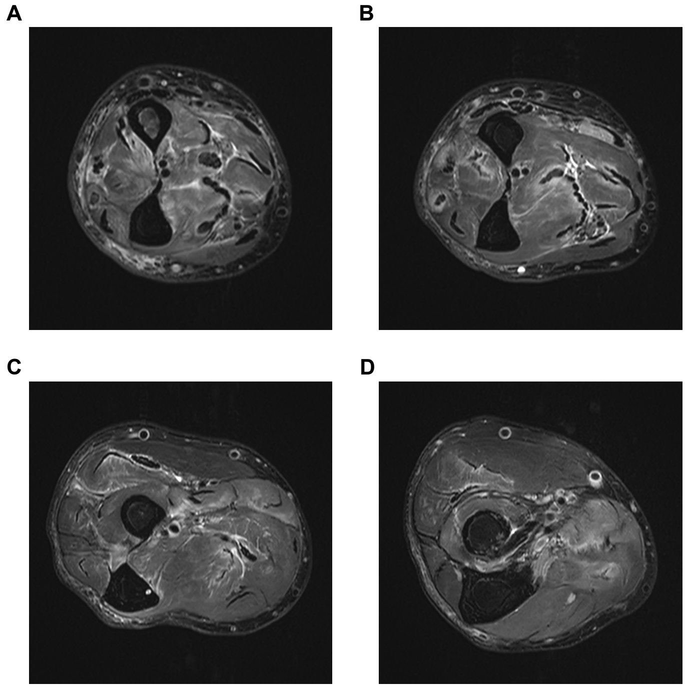

Fig. S37. MRI images of the subject's arm. The images of the right arm with MRI, the distances are 3.3 (**A**), 7.2 (**B**), 12 (**C**), 15 (**D**) cm from the elbow.

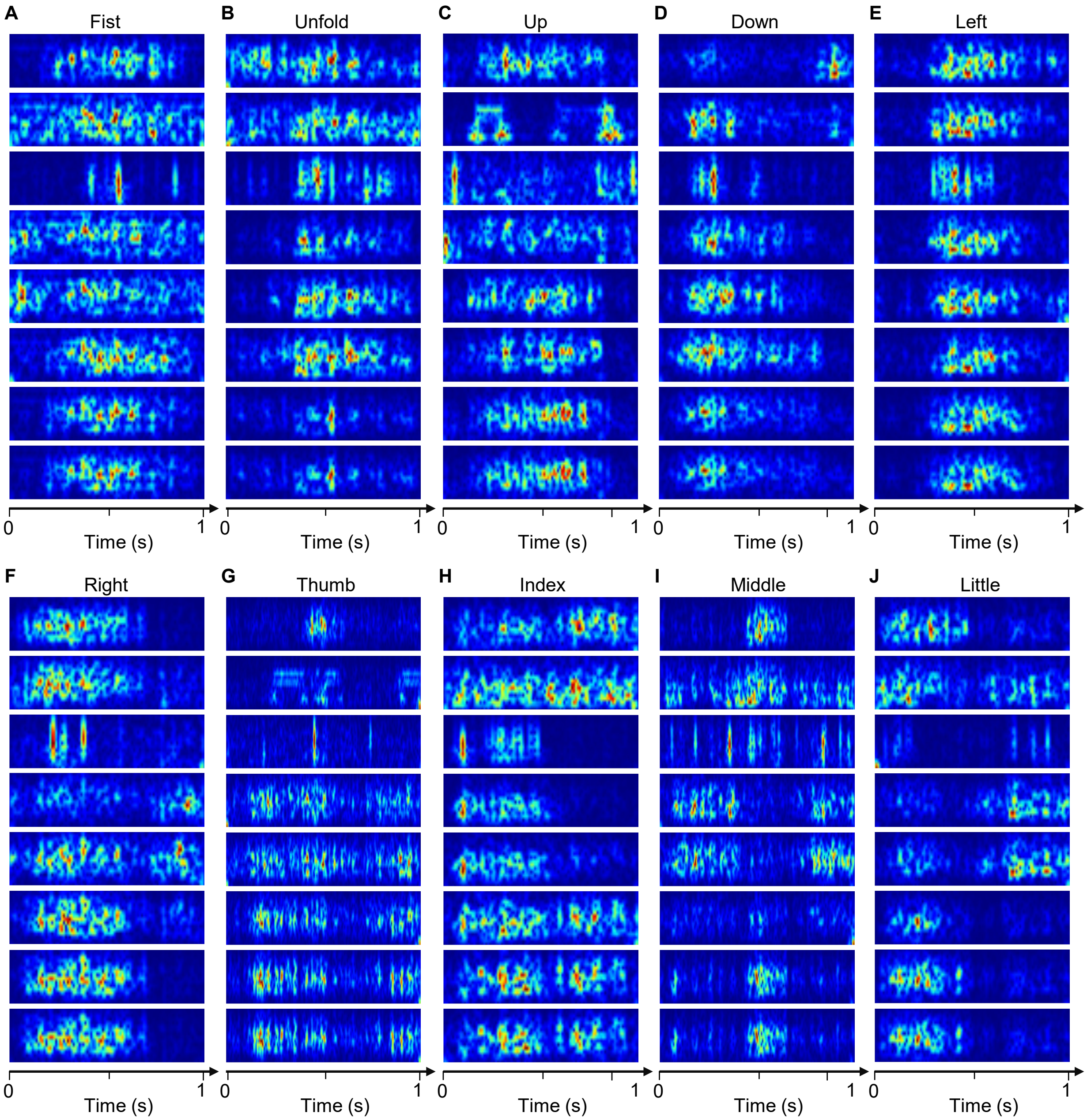

Fig. S38. Visualization of 8 channels data of all gestures from the subject. Time-frequence plots of fist (**A**), release (**B**), up (**C**), down (**D**), left (**E**), right (**F**) , thumb finger (**G**), index finger (**H**), middle finger (**I**), little finger (**J**) gestures. The frequence of each dependent signal was from 0 to 300 Hz.

Method	Recognized objects Number and Categories		Accuracy	Ref.
Thermal	11	Acrylic, Aluminum (Al), Brick, Cardboard, Glass, Medium-density fiberboard, Neoprene, Porcelain, Pine, Rubber, Steel	84%	(*55*)
Thermal	7	Plastic bag, Food, Carton, Can, Napkin, Orange Peel, Bread	94%	(*17*)
Triboelectric	4	Cotton, Paper, Copper, Resin	99.07%	(*56*)
Triboelectric	12	Al, Acrylic, Ethylene Vinyl Acetate (EVA), Glass, PU, PVC, Si, Wood, PET, Polystyrene (PS), PA66, PTFE	96.8%	(*57*)
Tactile (thermal)-visual	10	Crumpled paper, Cleaning cloth, Napkin, Plastic bag, Plastic bottle, Orange peel, Cup with cold water, Cup with alcohol, Cup with hot water, Empty cup	96.5%	(*23*)
Olfactory-tactile (force)	11	Orange, Towel, Arm, Stone, Can, Hair, Leg, Mouse, Worn Clothes, Mug, Carton	96.9%	(*58*)
Temperature-pressure	13	Al, Cu, Zn, Ti, Quartz, Acrylic, Polyethylene, Polypropylene, Paraffin wax, Rubber, Pine, Photosensitive resin, Corundum	95.9%	(*59*)
Piezoresistive-piezoelectric	8	EVA, Ecoflex, Sponge, PS, Wood, Foam, PDMS, Rubber	98.95%	(*60*)
Thermal-electrical	20	Cabbage, Glass, Fabric, Garlic, Single-crystal silicon, Polycrystalline silicon, Silicone rubber, Brass, Al, Charcoal, Pumpkin, Lemon, Conductive Gel, Polyethylene foam, Celery, Graphite, ABS, Stainless steel, Potato, Lamb,	98.03%	This work

Table S1. Comparison about multimodal sensing for material recognition.

REFERENCES AND NOTES

1. W. Wang, Y. Jiang, D. Zhong, Z. Zhang, S. Choudhury, J. Lai, H. Gong, S. Niu, X. Yan, Y. Zheng, C. C. Shih, R. Ning, Q. Lin, D. Li, Y. Kim, J. Kim, Y. Wang, C. Zhao, C. Xu, X. Ji, Y. Nishio, H. Lyu, J. B. H. Tok, Z. Bao, Neuromorphic sensorimotor loop embodied by monolithically integrated, low-voltage, soft e-skin. *Science* **380**, 735–742 (2023).

2. G. Gu, N. Zhang, H. Xu, S. Lin, Y. Yu, G. Chai, L. Ge, H. Yang, Q. Shao, X. Sheng, X. Zhu, X. Zhao, A soft neuroprosthetic hand providing simultaneous myoelectric control and tactile feedback. *Nat. Biomed. Eng.* **7**, 589–598 (2023).

3. Y. Yu, J. Nassar, C. Xu, J. Min, Y. Yang, A. Dai, R. Doshi, A. Huang, Y. Song, R. Gehlhar, A. D. Ames, W. Gao, Biofuel-powered soft electronic skin with multiplexed and wireless sensing for human-machine interfaces. *Sci. Robot.* **5**, eaaz7946 (2020).

4. Y. Luo, M. R. Abidian, J. H. Ahn, D. Akinwande, A. M. Andrews, M. Antonietti, Z. Bao, M. Berggren, C. A. Berkey, C. J. Bettinger, J. Chen, P. Chen, W. Cheng, X. Cheng, S. J. Choi, A. Chortos, C. Dagdeviren, R. H. Dauskardt, C. A. Di, M. D. Dickey, X. Duan, A. Facchetti, Z. Fan, Y. Fang, J. Feng, X. Feng, H. Gao, W. Gao, X. Gong, C. Guo, X. Guo, M. C. Hartel, Z. He, J. S. Ho, Y. Hu, Q. Huang, Y. Huang, F. Huo, M. M. Hussain, A. Javey, U. Jeong, C. Jiang, X. Jiang, J. Kang, D. Karnaushenko, A. Khademhosseini, D. Kim, I. Kim, D. Kireev, L. Kong, C. Lee, N. Lee, P. S. Lee, T. W. Lee, F. Li, J. Li, C. Liang, C. T. Lim, Y. Lin, D. Lipomi, J. Liu, K. Liu, N. Liu, R. Liu, Y. Liu, Y. Liu, Z. Liu, Z. Liu, X. J. Loh, N. Lu, Z. Lv, S. Magdassi, G. G. Malliaras, N. Matsuhisa, A. Nathan, S. Niu, J. Pan, C. Pang, Q. Pei, H. Peng, D. Qi, H. Ren, J. A. Rogers, A. Rowe, O. G. Schmidt, T. Sekitani, D. Seo, G. Shen, X. Sheng, Q. Shi, T. Someya, Y. Song, E. Stavrinidou, M. Su, X. Sun, K. Takei, X. Tao, B. C. K. Tee, A. V. Y. Thean, T. Q. Trung, C. Wan, H. Wang, J. Wang, M. Wang, S. Wang, T. Wang, Z. Wang, P. S. Weiss, H. Q. Wen, S. Xu, T. Xu, H. Yan, X. Yan, H. Yang, L. Yang, S. Yang, L. Yin, C. Yu, G. Yu, J. Yu, S. Yu, X. Yu, E. Zamburg, H. Zhang, X. Zhang, X. Zhang, X. Zhang, Y. Zhang, Y. Zhang, S. Zhao, X. Zhao, Y. Zheng, Y. Zheng, Z. Zheng, T. Zhou, B. Zhu, M. Zhu, R. Zhu, Y. Zhu, Y. Zhu, G. Zou, X. Chen, Technology roadmap for flexible sensors. *ACS Nano* **17**, 5211–5295 (2023).

5. S. Lin, J. Zhu, W. Yu, B. Wang, K. A. Sabet, Y. Zhao, X. Cheng, H. Hojaiji, H. Lin, J. Tan, C. Milla, R. W. Davis, S. Emaminejad, A touch-based multimodal and cryptographic bio-human-machine interface. *Proc. Natl. Acad. Sci. U.S.A.* **119**, e2201937119 (2022).

6. S. Sundaram, P. Kellnhofer, Y. Li, J. Zhu, A. Torralba, W. Matusik, Learning the signatures of the human grasp using a scalable tactile glove. *Nature* **569**, 698–702 (2019).

7. B. Hou, L. Yi, C. Li, H. Zhao, R. Zhang, B. Zhou, X. Liu, An interactive mouthguard based on mechanoluminescence-powered optical fibre sensors for bite-controlled device operation. *Nat. Electron.* **5**, 682–693 (2022).

8. Y. Lu, G. Yang, S. Wang, Y. Zhang, Y. Jian, L. He, T. Yu, H. Luo, D. Kong, Y. Xianyu, B. Liang, T. Liu, X. Ouyang, J. Yu, X. Hu, H. Yang, Z. Gu, W. Huang, K. Xu, Stretchable graphene-hydrogel interfaces for wearable and implantable bioelectronics. *Nat. Electron.* **7**, 51–65 (2024).

9. Y. Lu, D. Kong, G. Yang, R. Wang, G. Pang, H. Luo, H. Yang, K. Xu, Machine learning-enabled tactile sensor design for dynamic touch decoding. *Adv. Sci.* **10**, 2303949 (2023).

10. X. Guo, Z. Sun, Y. Zhu, C. Lee, Zero-biased bionic fingertip E-skin with multimodal tactile perception and artificial intelligence for augmented touch awareness. *Adv. Mater.* **36**, 2406778 (2024).

11. L. Scalco de Vasconcelos, Y. Yan, P. Maharjan, S. Kumar, M. Zhang, B. Yao, H. Li, S. Duan, E. Li, E. Williams, S. Tiku, P. Vidal, R. S. Solorzano-Vargas, W. Hong, Y. Du, Z. Liu, F. Iwane, C. Block, A. T. Repetski, P. Tan, P. Wang, M. G. Martín, J. del R. Millán, X. He, N. Lu, On-scalp printing of personalized electroencephalography e-tattoos. *Cell Biomater.* **1**, 100004 (2025).

12. Z. Huang, Y. Hao, Y. Li, H. Hu, C. Wang, A. Nomoto, T. Pan, Y. Gu, Y. Chen, T. Zhang, W. Li, Y. Lei, N. Kim, C. Wang, L. Zhang, J. W. Ward, A. Maralani, X. Li, M. F. Durstock, A. Pisano, Y. Lin, S. Xu, Three-dimensional integrated stretchable electronics. *Nat. Electron.* **1**, 473–480 (2018).

13. W. Heng, S. Yin, J. Min, C. Wang, H. Han, E. S. Sani, J. Li, Y. Song, H. B. Rossiter, W. Gao, A smart mask for exhaled breath condensate harvesting and analysis. *Science* **385**, 954–961 (2024).

14. L. E. Osborn, A. Dragomir, J. L. Betthauser, C. L. Hunt, H. H. Nguyen, R. R. Kaliki, N. V. Thakor, Prosthesis with neuromorphic multilayered e-dermis perceives touch and pain. *Sci. Robot.* **3**, eaat3818 (2018).

15. Y. Yan, Z. Hu, Z. Yang, W. Yuan, C. Song, J. Pan, Y. Shen, Soft magnetic skin for super-resolution tactile sensing with force self-decoupling. *Sci. Robot.* **6**, eabc8801 (2021).

16. Y. Yu, J. Li, S. A. Solomon, J. Min, J. Tu, W. Guo, C. Xu, Y. Song, W. Gao, All-printed soft human-machine interface for robotic physicochemical sensing. *Sci. Robot.* **7**, eabn0495 (2022).

17. G. Li, S. Liu, L. Wang, R. Zhu, Skin-inspired quadruple tactile sensors integrated on a robot hand enable object recognition. *Sci. Robot.* **5**, eabc8134 (2020).

18. M. Iskandar, A. Albu-Schäffer, A. Dietrich, Intrinsic sense of touch for intuitive physical human-robot interaction. *Sci. Robot.* **9**, eadn4008 (2024).

19. F. Liu, S. Deswal, A. Christou, M. S. Baghini, R. Chirila, D. Shakthivel, M. Chakraborty, R. Dahiya, Printed synaptic transistor-based electronic skin for robots to feel and learn. *Sci. Robot.* **7**, eabl7286 (2022).

20. K. Sim, Z. Rao, Z. Zou, F. Ershad, J. Lei, A. Thukral, J. Chen, Q. Huang, J. Xiao, C. Yu, Metal oxide semiconductor nanomembrane-based soft unnoticeable multifunctional electronics for wearable human-machine interfaces. *Sci. Adv.* **5**, eaav9653 (2019).

21. Y. Liu, C. K. Yiu, Z. Zhao, W. Park, R. Shi, X. Huang, Y. Zeng, K. Wang, T. H. Wong, S. Jia, J. Zhou, Z. Gao, L. Zhao, K. Yao, J. Li, C. Sha, Y. Gao, G. Zhao, Y. Huang, D. Li, Q. Guo, Y. Li, X. Yu, Soft, miniaturized, wireless olfactory interface for virtual reality. *Nat. Commun.* **14**, 2297 (2023).

22. A. Fleming, W. Liu, H. Huang, Neural prosthesis control restores near-normative neuromechanics in standing postural control. *Sci. Robot.* **8**, eadf5758 (2023).

23. Q. Mao, Z. Liao, J. Yuan, R. Zhu, Multimodal tactile sensing fused with vision for dexterous robotic housekeeping. *Nat. Commun.* **15**, 6871 (2024).

24. Y. Kim, E. Genevriere, P. Harker, J. Choe, M. Balicki, R. W. Regenhardt, J. E. Vranic, A. A. Dmytriw, A. B. Patel, X. Zhao, Telerobotic neurovascular interventions with magnetic manipulation. *Sci. Robot.* **7**, eabg9907 (2022).

25. M. Zhu, Z. Sun, Z. Zhang, Q. Shi, T. He, H. Liu, T. Chen, C. Lee, Haptic-feedback smart glove as a creative human-machine interface (HMI) for virtual/augmented reality applications. *Sci. Adv.* **6**, eaaz8693 (2020).

26. L. Tian, B. Zimmerman, A. Akhtar, K. Yu, M. Moore, J. Wu, R. J. Larsen, J. Lee, J. Li, Y. Liu, B. Metzger, S. Qu, X. Guo, K. E. Mathewson, J. Fan, J. Cornman, M. Fatina, Z. Xie, Y. Mao, J. Zhang, Y. Zhang, F. Dolcos, M. Fabiani, G. Gratton, T. Bretl, L. J. Hargrove, P. V. Braun, Y. Huang, J. A. Rogers, Large-area MRI-compatible epidermal electronic interfaces for prosthetic control and cognitive monitoring. *Nat. Biomed. Eng.* **3**, 194–205 (2019).

27. X. Liu, The more and less of electronic-skin sensors. *Science* **370**, 910–911 (2020).

28. Y. Li, K. Li, J. Chen, S. Wang, H. Lu, D. Wen, Pilot stress detection through physiological signals using a transformer-based deep learning model. *IEEE Sens. J.* **23**, 11774–11784 (2023).

29. S. Mao, E. Sejdić, A review of recurrent neural network-based methods in computational physiology. *IEEE Trans. Neural Netw. Learn Syst.* **34**, 6983–7003 (2023).

30. M. Perslev, M. Jensen, S. Darkner, P. Jennum, C. Igel, U-Time: A fully convolutional network for time series segmentation applied to sleep staging. *Adv. Neural Inf. Process. Syst.* **32**, (2019).

31. B. Rim, N.-J. Sung, S. Min, M. Hong, Deep learning in physiological signal data: A survey. *Sensors* **20**, 969 (2020).

32. K. Yi, Y. Wang, K. Ren, D. Li, Learning topology-agnostic EEG representations with geometry-aware modeling, *Adv. Neural Inf. Process. Syst.* **36**, 53875–53891 (2023).

33. A. Furui, S. Eto, K. Nakagaki, K. Shimada, G. Nakamura, A. Masuda, T. Chin, T. Tsuji, A myoelectric prosthetic hand with muscle synergy-based motion determination and impedance model-based biomimetic control. *Sci. Robot.* **4**, eaaw6339 (2019).

34. K. Kim, J. Hong, K. Bae, K. Lee, D. J. Lee, J. Park, H. Zhang, M. Sang, J. E. Ju, Y. U. Cho, K. Kang, W. Park, S. Jung, J. W. Lee, B. Xu, J. Kim, K. J. Yu, Extremely durable electrical impedance tomography-based soft and ultrathin wearable e-skin for three-dimensional tactile interfaces. *Sci. Adv.* **10**, eadr1099 (2024).

35. X. Yang, C. Forró, T. L. Li, Y. Miura, T. J. Zaluska, C. T. Tsai, S. Kanton, J. P. McQueen, X. Chen, V. Mollo, F. Santoro, S. P. Pasca, B. Cui, Kirigami electronics for long-term electrophysiological recording of human neural organoids and assembloids. *Nat. Biotechnol.* **42**, 1836–1843 (2024).

36. J. Liu, N. Liu, Y. Xu, M. Wu, H. Zhang, Y. Wang, Y. Yan, A. Hill, R. Song, Z. Xu, M. Park, Y. Wu, J. L. Ciatti, J. Gu, H. Luan, Y. Zhang, T. Yang, H. Y. Ahn, S. Li, W. Z. Ray, C. K. Franz, M. R. Macewan, Y. Huang, C. W. Hammill, H. Wang, J. A. Rogers, Bioresorbable shape-adaptive structures for ultrasonic monitoring of deep-tissue homeostasis. *Science* **383**, 1096–1103 (2024).

37. H. Ismail Fawaz, B. Lucas, G. Forestier, C. Pelletier, D. F. Schmidt, J. Weber, G. Webb, L. Idoumghar, P. A. Muller, F. Petitjean, InceptionTime: Finding AlexNet for time series classification, *Data Min. Knowl. Discov.* **34.6**, pp. 1936–1962 (2020).

38. K. K. Kim, M. Kim, K. Pyun, J. Kim, J. Min, S. Koh, S. E. Root, J. Kim, B. N. T. Nguyen, Y. Nishio, S. Han, J. Choi, C. Kim, J. B. H. Tok, S. Jo, S. H. Ko, Z. Bao, A substrate-less nanomesh receptor with meta-learning for rapid hand task recognition. *Nat. Electron.* **6**, 64–75 (2023).

39. A. Moin, A. Zhou, A. Rahimi, A. Menon, S. Benatti, G. Alexandrov, S. Tamakloe, J. Ting, N. Yamamoto, Y. Khan, F. Burghardt, L. Benini, A. C. Arias, J. M. Rabaey, A wearable biosensing system with in-sensor adaptive machine learning for hand gesture recognition. *Nat. Electron.* **4**, 54–63 (2021).

40. K. He, X. Zhang, S. Ren, J. Sun, "Deep residual learning for image recognition," in *Proceedings of the IEEE Conference on Computer Vision and Pattern Recognition* (2016), pp.770–778.

41. K. Ha, W. Zhang, H. Jang, S. Kang, L. Wang, P. Tan, H. Hwang, N. Lu, Highly sensitive capacitive pressure sensors over a wide pressure range enabled by the hybrid responses of a highly porous nanocomposite. *Adv. Mater.* **33**, 2103320 (2021).

42. R. Yang, A. Dutta, B. Li, N. Tiwari, W. Zhang, Z. Niu, Y. Gao, D. Erdely, X. Xin, T. Li, H. Cheng, Iontronic pressure sensor with high sensitivity over ultra-broad linear range enabled by laser-induced gradient micro-pyramids. *Nat. Commun.* **14**, 2907 (2023).

43. H. Yuk, B. Lu, S. Lin, K. Qu, J. Xu, J. H. Luo, X. Zhao, 3D printing of conducting polymers. *Nat. Commun.* **11**, 1604 (2020).

44. F. Ilievski, A. D. Mazzeo, R. E. Shepherd, X. Chen, G. M. Whitesides, Soft robotics for chemists. *Angew. Chem. Int. Ed. Engl.* **50**, 1890–1895 (2011).

45. W. Tang, Y. Zhong, H. Xu, K. Qin, X. Guo, Y. Hu, P. Zhu, Y. Qu, D. Yan, Z. Li, Z. Jiao, X. Fan, H. Yang, J. Zou, Self-protection soft fluidic robots with rapid large-area self-healing capabilities. *Nat. Commun.* **14**, 6430 (2023).

46. H. Zhao, K. O'Brien, S. Li, R. F. Shepherd, Optoelectronically innervated soft prosthetic hand via stretchable optical waveguides. *Sci. Robot.* **1**, eaai7529 (2016).

47. C. Jiang, D. Wang, B. Zhao, Z. Liao, G. Gu, Modeling and inverse design of bio-inspired multi-segment pneu-net soft manipulators for 3D trajectory motion. *Appl. Phys. Rev.* **8**, 041416 (2021).

48. S. Varma, R. Simon, Bias in error estimation when using cross-validation for model selection. *BMC Bioinformatics* **7**, 91 (2006).

49. S. Bates, T. Hastie, R. Tibshirani, Cross-validation: What does it estimate and How well does it do it? *J. Am. Stat. Assoc.* **119**, 1434–1445 (2024).

50. N. Jarrassé, C. Nicol, F. Richer, A. Touillet, N. Martinet, J. Paysant, J. B. De Graaf, "Voluntary phantom hand and finger movements in transhumeral amputees could be used to naturally control polydigital prostheses," in *2017 International Conference on Rehabilitation Robotics* (2017), pp. 1239–1245.

51. X. Wang, J. Wang, N. Fei, D. Duanmu, B. Feng, X. Li, W.-Y. Ip, Y. Hu, Alternative muscle synergy patterns of upper limb amputees. *Cogn. Neurodyn.* **18**, 1119–1133 (2024).

52. K. T. Reilly, C. Mercier, M. H. Schieber, A. Sirigu, Persistent hand motor commands in the amputees' brain. *Brain* **129**, 2211–2223 (2006).

53. X. Li, O. W. Samuel, X. Zhang, H. Wang, P. Fang, G. Li, A motion-classification strategy based on sEMG-EEG signal combination for upper-limb amputees. *J. Neuroeng. Rehabil.* **14**, 2 (2017).

54. C. Lin, X. Niu, J. Zhang, X. Fu, Improving motion intention recognition for trans-radial amputees based on sEMG and transfer learning. *Appl. Sci.* **13**, 11071 (2023).

55. T. Bhattacharjee, J. Wade, C. C. Kemp, in Robotics: Science and Systems (2015), vol. 2015.

56. Z. Song, J. Yin, Z. Wang, C. Lu, Z. Yang, Z. Zhao, Z. Lin, J. Wang, C. Wu, J. Cheng, Y. Dai, Y. Zi, S.-L. Huang, X. Chen, J. Song, G. Li, W. Ding, A flexible triboelectric tactile sensor for simultaneous material and texture recognition. *Nano Energy* **93**, 106798 (2022).

57. X. Qu, Z. Liu, P. Tan, C. Wang, Y. Liu, H. Feng, D. Luo, Z. Li, Z. L. Wang, Artificial tactile perception smart finger for material identification based on triboelectric sensing. *Sci. Adv.* **8**, eabq2521 (2022).

58. M. Liu, Y. Zhang, J. Wang, N. Qin, H. Yang, K. Sun, J. Hao, L. Shu, J. Liu, Q. Chen, P. Zhang, T. H. Tao, A star-nose-like tactile-olfactory bionic sensing array for robust object recognition in non-visual environments. *Nat. Commun.* **13**, 79 (2022).

59. W. Yang, M. Xie, X. Zhang, X. Sun, C. Zhou, Y. Chang, H. Zhang, X. Duan, Multifunctional soft robotic finger based on a nanoscale flexible temperature-pressure tactile sensor for material recognition. *ACS Appl. Mater. Interfaces* **13**, 55756–55765 (2021).

60. Y. Qiu, S. Sun, X. Wang, K. Shi, Z. Wang, X. Ma, W. Zhang, G. Bao, Y. Tian, Z. Zhang, H. Ding, H. Chai, A. Liu, H. Wu, Nondestructive identification of softness via bioinspired multisensory electronic skins integrated on a robotic hand. *Npj Flex. Electron.* **6**, 45 (2022).
